# Identification of MicroRNA-Like RNAs in Mycelial and Yeast Phases of the Thermal Dimorphic Fungus *Penicillium marneffei*

**DOI:** 10.1371/journal.pntd.0002398

**Published:** 2013-08-22

**Authors:** Susanna K. P. Lau, Wang-Ngai Chow, Annette Y. P. Wong, Julian M. Y. Yeung, Jessie Bao, Na Zhang, Si Lok, Patrick C. Y. Woo, Kwok-Yung Yuen

**Affiliations:** 1 State Key Laboratory of Emerging Infectious Diseases, The University of Hong Kong, Hong Kong, China; 2 Research Centre of Infection and Immunology, The University of Hong Kong, Hong Kong, China; 3 Carol Yu Centre for Infection, The University of Hong Kong, Hong Kong, China; 4 Department of Microbiology, The University of Hong Kong, Hong Kong, China; 5 Genome Research Centre, The University of Hong Kong, Hong Kong, China; University of California San Diego School of Medicine, United States of America

## Abstract

**Background:**

*Penicillium marneffei* is the most important thermal dimorphic fungus causing systemic mycosis in China and Southeast Asia. While miRNAs are increasingly recognized for their roles in post-transcriptional regulation of gene expression in animals and plants, miRNAs in fungi were less well studied and their potential roles in fungal dimorphism were largely unknown. Based on *P. marneffei* genome sequence, we hypothesize that miRNA-like RNAs (milRNAs) may be expressed in the dimorphic fungus.

**Methodology/Principal Findings:**

We attempted to identify milRNAs in *P. marneffei* in both mycelial and yeast phase using high-throughput sequencing technology. Small RNAs were more abundantly expressed in mycelial than yeast phase. Sequence analysis revealed 24 potential milRNA candidates, including 17 candidates in mycelial and seven in yeast phase. Two genes, *dcl-1* and *dcl-2*, encoding putative Dicer-like proteins and the gene, *qde-2*, encoding Argonaute-like protein, were identified in *P. marneffei*. Phylogenetic analysis showed that *dcl-2* of *P. marneffei* was more closely related to the homologues in other thermal dimorphic pathogenic fungi than to *Penicillium chrysogenum* and *Aspergillus* spp., suggesting the co-evolution of *dcl-2* among the thermal dimorphic fungi. Moreover, *dcl-2* demonstrated higher mRNA expression levels in mycelial than yeast phase by 7 folds (*P*<0.001). Northern blot analysis confirmed the expression of two milRNAs, *PM-milR-M1* and *PM-milR-M2*, only in mycelial phase. Using *dcl-1^KO^*, *dcl-2^KO^*, *dcl^DKO^* and *qde-2^KO^* deletion mutants, we showed that the biogenesis of both milRNAs were dependent on *dcl-2* but not *dcl-1* or *qde-2*. The mRNA expression levels of three predicted targets of *PM-milR-M1* were upregulated in knockdown strain *PM-milR-M1* ^KD^, supporting regulatory function of milRNAs.

**Conclusions/Significance:**

Our findings provided the first evidence for differential expression of milRNAs in different growth phases of thermal dimorphic fungi and shed light on the evolution of fungal proteins involved in milRNA biogenesis and possible role of post-transcriptional control in governing thermal dimorphism.

## Author Summary

*Penicillium marneffei* is the most important thermal dimorphic pathogenic fungus in Southeast Asia. Despite findings on diverse genes and mechanisms involved in dimorphic switching, the key to signally pathways governing the switch is still unknown. Since miRNAs are important regulatory molecules in eukaryotes, we attempt to define if miRNAs are expressed in different growth phases of *P. marneffei*. Using high-throughput sequencing, we identified 24 potential milRNA candidates in *P. marneffei*, which were more abundantly expressed in mycelial than yeast phase. Two genes, *dcl-1* and *dcl-2*, encoding Dicer-like proteins and the gene, *qde-2*, encoding Argonaute-like protein, were also identified. Phylogenetic analysis showed that *dcl-2* of *P. marneffei* was more closely related to the homologues in other thermal dimorphic pathogenic fungi than to *Penicillium chrysogenum* and *Aspergillus* spp.. *dcl-2* demonstrated higher mRNA levels in mycelial than yeast phase. Northern blot analysis confirmed expression of two milRNAs, *PM-milR-M1* and *PM-milR-M2*, only in mycelial phase, whose expression was dependent on *dcl-2* but not *dcl-1* or *qde-2*. The mRNA levels of three predicted targets of *PM-milR-M1* were upregulated in knockdown strain *PM-milR-M1* ^KD^, supporting its regulatory function. This study represents the first discovery of milRNAs in thermal dimorphic fungi, with differential expression in different growth phases.

## Introduction

*Penicillium marneffei* is the most important thermal dimorphic fungus causing respiratory, skin and systemic mycosis in Southeast Asia [1]–[4]. Recently, it has been renamed as *Talaromyces* based on phylogenetic analyses [5]. The fungus was first discovered in Chinese bamboo rats, *Rhizomys sinensis*, and subsequently isolated from other species of bamboo rats [6], [7]. While only 18 cases of human diseases were reported until 1985 [8], the emergence of the HIV pandemic in the 1980's has resulted in increasing reports of HIV-associated *P. marneffei* infections in Southeast Asia where the fungus is endemic. Penicilliosis is the third most common indicator disease of AIDS In northern Thailand [2]. In Hong Kong, about 10% of HIV patients are infected with *P. marneffei*, which represents the sixth leading cause of death [9], [10]. Cases of imported *P. marneffei* infections have also been reported from countries outside Southeast Asia [11], [12]. In addition, *P. marneffei* infections are increasingly reported in other immunocompromised patients, such as transplant recipients and others on immunosuppressant therapy [13]–[16]. Despite its medical importance, the mode of transmission, and dimorphic and pathogenic mechanisms of *P. marneffei* remain largely unknown. In particular, *P. marneffei* exhibits distinct cellular morphologies in different temperatures, in mycelial phase at 25°C and yeast phase at 37*°*C. During the mycelial phase, hyphae can differentiate to produce conidia which are believed to be the infectious form being inhaled to the lungs of infected hosts. When these conidia are phagocytosed by alveolar macrophages, they germinate into yeast cells as the tissue form. Despite the efforts of using various gene knockout experiments in identifying diverse genes and complex mechanisms involved in dimorphic switching in *P. marneffei*, the signals that trigger the switch in response to temperature and signaling pathways leading to the transition remain elusive [17].

MicroRNAs or miRNAs are small non-coding endogenous RNAs of approximately 22 nt, which play important roles in post-transcriptional regulation of gene expression in animals and plants [18]. They are now known to comprise one of the most abundant classes of gene regulatory molecules in multicellular organisms. The mature miRNAs negatively regulate gene expression by targeting mRNAs mediated through complementary binding to the open-reading frame or untranslated (UTR) regions of specific target genes. Interactions with targets can be through imprecise base pairing leading to translational inhibition in animals, or near-perfect complementarity leading to mRNA cleavage in plants [19], [20]. In animals, miRNAs have been shown to play various roles in ranging from cell development, proliferation and differentiation, apoptosis, carcinogenesis to immunity [21]–[23]. In plants, they are also involved in plant development, stress response and antibacterial resistance [24], [25], [26].

The first known miRNA *lin-4* was discovered in *Caenorhabditis elegans* in 1993 [27]. However, it was only until 2000 that the second miRNA, *let-7*, also in *C. elegans*, was identified [28]. With the advent of molecular and bioinformatics tools, numerous miRNAs have now been identified in animals, plants, viruses and unicellular organisms, with >25,000 miRNAs being currently included in the miRNA database, miRBase release 19.0 [29]. Although small RNA pathways have been found in various fungi, the existence of miRNAs and their roles in fungi has been less well understood. Recently, miRNA-like small RNAs (milRNAs) have been identified in the red bread mold, *Neurospora crassa*, the plant pathogenic fungus, *Sclerotinia sclerotiorum*, the entomopathogenic fungus, *Metarhizium anisopliae* and the human pathogenic yeast, *Cryptococcus neoformans* [30]–[33]. However, their existence in thermal dimorphic fungi and potential roles in fungal dimorphism were largely unknown.

In 2002, we started the *P. marneffei* genome project in an attempt to expedite the study of biology, epidemiology and virulence factors of this dimorphic fungus [34]–[41]. Based on the available genome sequence data, potential genes encoding proteins important for miRNA biogenesis can be identified in *P. marneffei*. Since miRNAs are important gene regulatory molecules in multicellular organisms, we hypothesize that milRNAs may be expressed in *P. marneffei* and involved in the regulation of thermal dimorphism. We attempted to identify milRNAs in *P. marneffei* in both mycelial and yeast phase using high-throughput Illumina DNA sequencing. Sequence analysis revealed 24 potential milRNA candidates, which were more abundantly expressed in mycelial than yeast phase of *P. marneffei*. Two genes, *dcl-1* and *dcl-2*, encoding putative Dicer-like proteins and the gene, *qde-2*, encoding quelling-deficient-2, an Argonaute-like protein, were also identified. Northern blot analysis confirmed the differential expression of two milRNAs, *PM-milR-M1* and *PM-milR-M2*, in mycelial phase, which was dependent on *dcl-2* but not *dcl-1* or *qde-2*. Our findings provided evidence for the existence of milRNAs in thermal dimorphic fungi and differential expression of milRNAs during different growth phases, which may provide new insights into the mechanism governing thermal dimophism.

## Materials and Methods

### Ethics statement

*P. marneffei* strain PM1 was obtained from an already-existing collection from the clinical microbiology laboratory in Queen Mary Hospital and the strain was anonymized.

### *P. marneffei* strains and growth conditions

*P. marneffei* strain PM1 was isolated from a patient with culture-documented penicilliosis in Hong Kong. Knock-out mutant strains including *dcl-1^KO^*, *dcl-2^KO^*, *dcl-1 dcl-2* double mutant (*dcl^DKO^*) and *qde-2^KO^* were generated as described below. All *P. marneffei* strains were grown on Sabouraud dextrose agar (SDA) (Oxoid, Cambridge, UK) at 25°C for 7 days for the collection of conidia as described previously [40]. Conidia were collected by scraping and resuspension in 0.1% Tween-20 with PBS followed by three washes in sterile PBS before subculturing into liquid cultures in BHI medium (Difco, NJ, USA) in a shaker at 37°C for yeasts or at 25°C for mycelia for 48 hours. Cells were enumerated using a hemocytometer.

### Small RNA purification, library preparation and sequencing

Small RNA libraries were constructed for mycelial and yeast phases of *P. marneffei*. Total RNAs were extracted from cultures grown at the respective phase using Plant Isolation Aid (Ambion, Austin, TX, USA) and mirVana miRNA Isolation Kit (Ambion) after mechanical disruption with acid-washed glass beads (Sigma-Aldrich, Missouri, USA), and treated with DNA-free Kit (Ambion) to remove residual DNA. 10 µg of total RNAs from both mycelial and yeast phases was treated with RiboMinus Eukaryote Kit for RNA-Seq (Invitrogen, Carlsbad, CA) to remove ribosomal RNAs (rRNAs). The rRNA-depleted RNA was concentrated by ethanol precipitation in the presence of glycogen carrier (Ambion). RNA concentration was determined using a NanoDrop ND-1000 spectrophotometer (NanoDrop Technologies, Wilmington, DE). A strand-specific library construction protocol was used to generate template for Illumina DNA sequencing [42]. An adenylated 3′- adaptor (Integrated DNA Technologies, Coralville, IA) was first ligated to the 3′ ends of a small RNA (≤60 nt) fraction was extracted from 15% denaturing polyacrylamide gel. The 3′ adaptor-ligated small RNAs were then ligated with a 5′-adaptor (Integrated DNA Technologies). Adaptor-ligated small RNAs were reverse transcribed into first-strand cDNA using a primer hybridizing to the 3′-adaptor using SuperScript II reverse transcriptase (Invitrogen). First strand cDNA was amplified by polymerase chain reaction (PCR) from Illumina/Solexa PCR primer binding sites present on the 5′- and 3′-adaptors to generate templates for sequencing on the Illumina Genome Analyzer IIx (Illumina, San Diego, CA).

### Small RNA analyses

Sequence reads were processed to remove low quality reads, adaptor and adaptor-dimer sequences, and nuclear and mitrochondrial rRNA sequences to yield 16,479,305 filtered reads for mycelial and 12,754,677 filtered reads for yeast phase respectively. Relative expression levels were estimated by normalizing read counts for each non-redundant small RNA species against RPM (number of reads per million mapped reads) as mapped to the draft *P. marneffei* PM1 genome sequence [39]. Small RNA sequences between 17–30 nt were selected to identify perfect matches to the genome using Bowtie (0.12.8) [43].

### Identification of milRNAs and milRNA loci

To identify milRNA candidates, other non-coding RNAs including rRNAs and tRNAs were first excluded. Potential milRNA candidates were predicted with miRDeep [44] based on draft *P. marneffei* PM1 genome. Analysis was performed with the following adjustments: (1) Filtering ubiquitous alignments, keeping only reads that were perfectly mapped to no more than 5 different regions in the genome; (2) Potential precursor sequences were excised from the genome with the size of 250 nt flanking to the sequencing reads; and (3) Hybridization temperatures of 25°C and 37°C were used in the script regarding RNAfold for deep sequencing data from mycelial and yeast form of *P. marneffei* respectively. milRNA candidates were identified with the following criteria: small RNAs that formed a stem-loop structure (hairpin) with flanking sequences (up to 250 nt), as examined by RNAfold in miRDeep package.

### Identification and sequencing analysis of *dcl-1*, *dcl-2* and *qde-2* genes in *P. marneffei*

Based on the predicted protein sequences of corresponding genes from *N. crassa*, putative *dcl-1*, *dcl-2* and *qde-2* genes in the *P. marneffei* strain PM1 draft genome sequence (GenBank accession no. AGCC00000000) were searched using BLASTP algorithm. Introns were predicted by performing pairwise alignment with the annotated *Talaromyces stipitatus* (teleomorph of *Penicillium emmonsii*) (GenBank accession no. ABAS00000000) and *P. marneffei* strain ATCC 18224 (GenBank accession no. ABAR00000000) genome sequences. The complete coding sequences of *dcl-1*, *dcl-2* and *qde-2* of *P. marneffei* were PCR amplified from cDNA using primers derived from *P. marneffei* genome sequence as described previously (Table 1) [36]. To perform phylogenetic analysis, putative *dcl-1*, *dcl-2* and *qde-2* homologues from representative fungal species were retrieved using BLASTP against the GenBank database. Nucleotide sequences of the internal transcribed spacer (ITS) regions were obtained from GenBank. Phylogenetic trees were constructed using the maximum-likelihood method with 1000 bootstrap replicates with Mega 5.1 [45]. WAG+F+G (for *dcl-1* and *dcl-2*) and rtREV+G (for *qde-2*) amino acid substitution models, K2+G nucleotides substitution models (for ITS) with 5 gamma categories were used. Nine-hundred and fourteen, 764 and 525 amino acid positions of *dcl-1*, *dcl-2* and *qde-2* respectively, and 465 nucleotides positions of ITS, were used for analysis. Domains were predicted using the Conserved Domains Database of NCBI and PFAM (http://pfam.sanger.ac.uk/search?tab=searchSequenceBlock) and manual inspection of multiple alignments with homologous sequences.

**Table 1:** Primers used in this study.

Gene Targets	Primers	Purpose
Upstream of *dcl-1*	LPW10929 5′-GAAGATCTCCGTAGTGCTTCTGATTGGTCTGAG-3′	pAN7-1 cloning
	LPW10930 5′-GAAGATCTTCTTTTGCGGCCTTTGTAAGTCTG-3′	(*Bgl*II and *Hind*III)
Downstream of *dcl-1*	LPW10931 5′-TGATTGAAGATCCTCCCAAGGTTG-3′	
	LPW10932 5′-CCCAAGCTTGGGTGGTTCGTGAGATAGGTGGTGGATA -3′	
Upstream of *dcl-2*	LPW13339 5′-GAAGATCTCGCCGAACAAACGGAAGAAGGAGA-3′	pAN7-1 cloning
	LPW13340 5′-TGGCTTCTCCGAAGCTCTCTATGG-3′	(*Bgl*II and *Sfo*I)
Downstream of *dcl-2*	LPW13341 5′-ATAGGCGCCCCTAGTCGATTTTCATGAACGGACC-3′	
	LPW13342 5′-ATAGGCGCCGGATTACATAACATACCGTCGGCTG-3′	
Upstream of *qde-2*	LPW12475 5′-ACCCAATAAGGATGAGGAAGTTCGG-3′	pAN7-1 cloning
	LPW12476 5′-GAAGATCT AAGTCAGTCGCAATCTCGTCCCG-3′	(*Bgl*II and *Sbf*I)
Downstream of *qde-2*	LPW12718 5′-GACCTGCAGGACACATCACCAAGTGAAGTGTCAC-3′	
	LPW12719 5′-GACCTGCAGGATCCGCTTGACTCCAGGTGGTA -3′	
Upstream of *dcl-1*	LPW10929 5′-GAAGATCTCCGTAGTGCTTCTGATTGGTCTGAG-3′	pAN8-1 cloning
	LPW10930 5′-GAAGATCTTCTTTTGCGGCCTTTGTAAGTCTG-3′	(*Bgl*II and *Sfo*I)
Downstream of *dcl-1*	LPW12799 5′-ATAGGCGCCTGATTGAAGATCCTCCCAAGGTTG-3′	
	LPW12800 5′-ATAGGCGCCTGGTTCGTGAGATAGGTGGTGGATA -3′	
*dcl-1*	LPW13343 5′-TTTACGGGACGTAAATGGCGGCCTA-3′	qPCR
	LPW13344 5′-AATTCTAGGCGCTGGTAAGTCGGC-3′	
	LPW21945 5′-ATGTTGGAGACTCTTACCCTGG-3′	cDNA amplification
	LPW22072 5′-CTCGGCATTCCATAGTTTGT-3′	& sequencing
	LPW22073 5′-GCCGAGGTTTCATGGAAAGA-3′	
	LPW22074 5′-CGGTTCAGCTGGAGAAAACA-3′	
	LPW22075 5′-CGTCAAACCTTCATTCTGGA-3′	
	LPW22076 5′-CGGTGTTGAATAAATTCCTG-3′	
	LPW22077 5′-ATATCTTTATTCGTTGGAAGTCCG-3′	
	LPW21946 5′-TACATCACGGGACTCGGGGA-3′	
	LPW21943 5′-ATGGCCATAGAGAGCTTCGG-3′	
	LPW22066 5′-TCGGCCAAAACGTCCCTTTG-3′	
	LPW22067 5′-CTCATCTCTTGAGCGTGCAC-3′	
*dcl-2*	LPW13347 5′-GTGTGAAGTGATATTGCCAAAGGG-3′	qPCR
	LPW13348 5′-CATTTTGTAACGGTTCAGCTGGAG-3′	
	LPW22068 5′-CGATGATGAATGGTCGTGAA-3′	cDNA amplification
	LPW22069 5′-GATGCAAAATCTTGGAATGG-3′	& sequencing
	LPW22070 5′-GCATCAGGTGCATTTCTTGG-3′	
	LPW22071 5′-CGATTCCTTGCTAGACCACTTCG-3′	
	LPW21944 5′-CACTGCCGGCTTGACTGTCC-3′	
	LPW21947 5′-ATGTCCAGCGGGTATAGACG-3′	
	LPW22078 5′-TCTAGCCGAGCCTTGCCCTT-3′	
	LPW22079 5′-CTAGTACAGCCTCGGTAGACA-3′	
	LPW22080 5′-ACCTTCAGAGACTCCATCGC-3′	
*qde-2*	LPW14804 5′-GCCTCATCAAAATCCCCGGT-3′	qPCR
	LPW14805 5′-GGAGAAACGACGACACCCAT-3′	
	LPW22081 5′-CCGCCCTTCCTGAGAACATT-3′	cDNA amplification
	LPW22082 5′-TTAGATATAGAACATCGTGT-3′	& sequencing
*Actin*	LPW20631 5′-GAACGTGAAATCGTCCGT-3′	qPCR
	LPW20160 5′-AGCAAGAATGGAACCACC-3′	
*PM-milR-M1* gene	LPW20742 5′-CCGCTCGAGGACGCACAAAACAATGCAAA-3′	pSilent-1 cloning
locus	LPW20743 5′-CCCAAGCTTGGGGTATTGCCGTTGATCCATCG-3′	(*Xho*I-*Hin*dIII)
	LPW20740 5′-GGGGTACCGACGCACAAAACAATGCAAA-3′	pSilent-1 cloning
	LPW20741 5′-GAAGATCTGTATTGCCGTTGATCCATCG-3′	(*Bgl*II-*Kpn*I)
	LPW23656 5′- TTGCCAATAACAAAGACTCTTC-3′	qPCR
	LPW23657 5′- TCTTAGTCATAGCATCTGCG-3′	
*RanBP10*	LPW23241 5′- CAAGTGCTGGCACAGGTCTA-3′	qPCR
	LPW23242 5′- TATATCCCAGCTTCACGCGG-3′	
Cyctochrome P450	LPW23438 5′- GACGGCTATCAGTCTCACGG-3′	qPCR
	LPW23439 5′- GAGGCCGAACGGCATATACA-3′	
Conserved	LPW23499 5′- TGTCGGCAACCATGTGCTAT-3′	qPCR
hypothetical protein	LPW23500 5′- CATTTCTTCGATGCAGGCGG-3′	

### Construction of *dcl-1^KO^*, *dcl-2^KO^*, *dcl^DKO^* and *qde-2^KO^* mutants of *P. marneffei*

**Figure 1:**
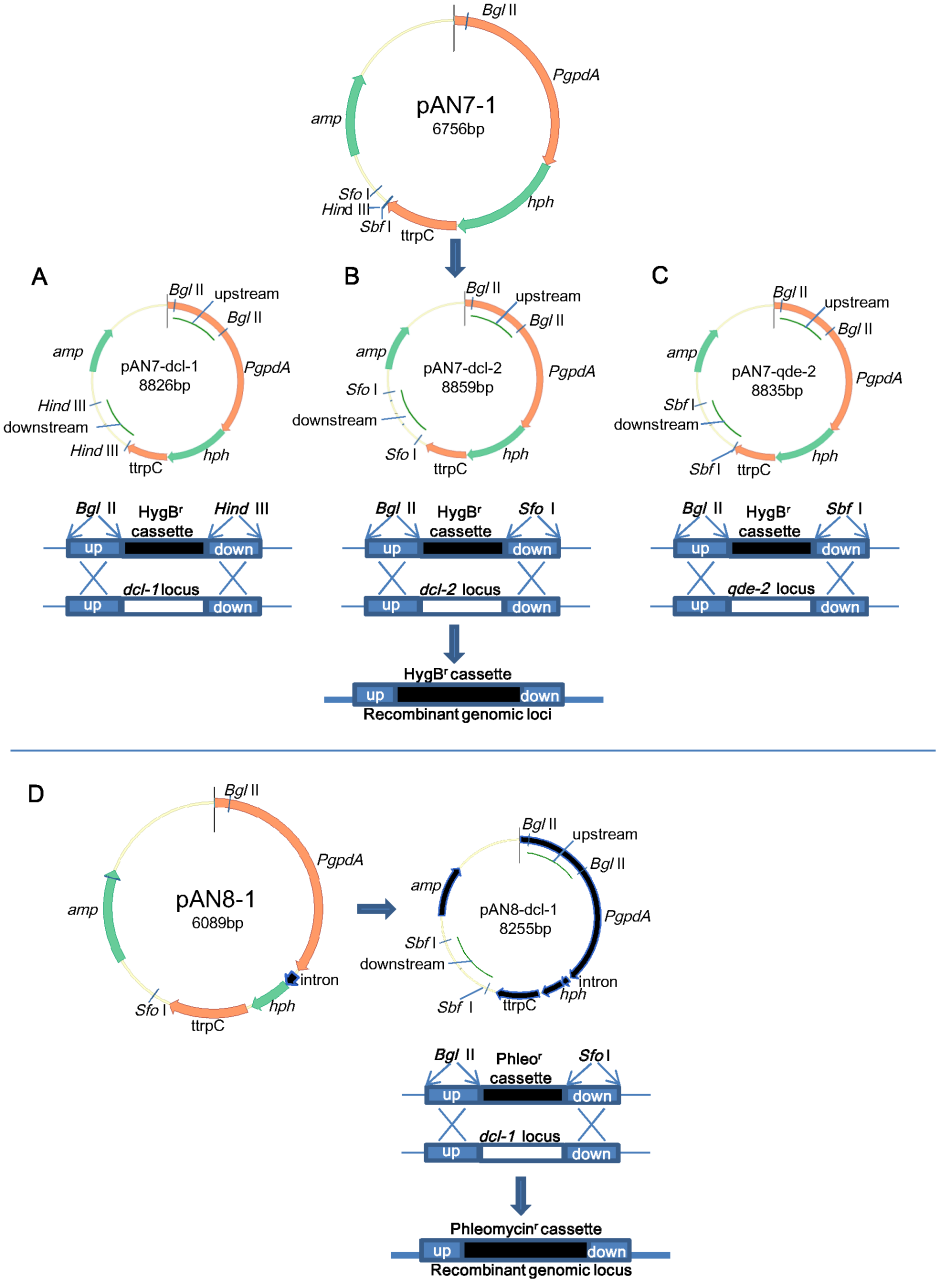
Deletion of (A) *dcl-1*, (B) *dcl-2*, (C) *qde-2*, (D) *dcl-1*/*dcl-2* in *P. marneffei* by homologous recombination. Plasmids pAN7-1 and pAN8-1 were used to construct the knockout plasmids of pAN7-*dcl-1*, pAN7-*dcl-2*, pAN7-*qde-2* and pAN8-*dcl-1* respectively.

Deletion mutants were generated by homologous recombination (Fig. 1). Based on *dcl-1*, *dcl-2* and *qde-2* gene sequences from *P. marneffei* strain PM1, primers were designed to amplify upstream and downstream fragments of *dcl-1*, *dcl-2* and *qde-2* for the construction of the corresponding knockout constructs using the vector pAN7-1 (a gift from Dr. P. J. Punt) as described previously [46], [47]. The flanking sequences upstream and downstream of *dcl-1*, *dcl-2* and *qde-2* were amplified by PCR using DNA extracted from strain PM1 with primers shown in Table 1. PCR products of upstream and downstream flanking fragments were ligated into corresponding restriction sites of plasmid pAN7-1 to generate the knock-out plasmids pAN7-*dcl-1*, pAN7-*dcl-2* and pAN7-*qde-2* as shown in Fig. 1. The resultant plasmids were linearized with *Ahd*I and transformed to strain PM1 according to previous publications [40], [48]. SDA supplemented with 150 µg/ml hygromycin B was used as selection medium. To construct *dcl^DKO^*, PCR products of *dcl-1* flanking fragments were ligated into vector pAN8-1 (a gift from Dr. P. J. Punt) as described previously [46], [49] to generate the knock-out plasmid pAN8-*dcl-1* (Fig. 1). pAN8-*dcl-1* was linearized with *Ahd*I and transformed to *dcl-2^KO^* to generate the double mutant *dcl^DKO^*, using SDA supplemented with 100 µg/ml phleomycin as selection medium.

### Northern blot analyses

Northern blot analysis was performed according to published protocols with modifications [30], [50]. Briefly, 10–20 µg of small RNAs was separated on 12% denaturing polyacrylamide gel and transferred onto a positively charged nylon membrane (Amersham Biosciences, United Kingdom) with NorthernMax Transfer buffer (Ambion) by means of capillary force for 1 h. Crosslinking of RNA to Hybond-NX was performed using a CL-1000 UV Cross-linker (UVP) according to the manufacturer's instructions, followed by baking at 80°C for 2 h. Hybridization was performed in ULTRAhyb-Oligo hybridization buffer (Ambion) for 3′ digoxigenin (DIG) labeled RNA probes (Sigma-Aldrich). Detection of the DIG-labeled probe on the blot was performed by using DIG Luminescent Detection kit (Roche).

### Target prediction for milRNAs

The potential targets of milRNA candidates were predicted using the predicted gene sequences, including their 5′ and 3′ UTRs, of the *P. marneffei* strain PM1 and ATCC strain 18442 draft genomes by the RNAhybrid program [51] with or without mismatches or insertions at positions 9–11 of the milRNA and with parameters that encourage complete complementarity at the seed region (positions 2–7 of the milRNA) [52].

### Construction of *PM-milR-M1* gene knockdown plasmid of *P. marneffei*

To knockdown the *PM-milR-M1* gene locus, plasmid pSilent-1 [53], obtained from the Fungal Genetics Stock Center, was used to construct the pSilent-M1 plasmid as previously described [40]. Briefly, the internal fragments (sense and antisense) were amplified with primers shown in Table 1 and cloned into the *Xho*I-*Hin*dIII and *Bgl*II-*Kpn*I sites of pSilent-1 plasmid, resulting in pSilent-M1. The wild type *P. marneffei* strain PM1 was transformed with linearized pSilent-M1, using 200 µg/ml hygromycin for selection.

### Quantitative real-time RT-PCR

Total RNA was extracted using RiboPure-Yeast (Ambion). Reverse transcription was performed using the SuperScript III kit (Invitrogen). Real-time RT-PCR assays were performed as described previously with modifications [36], using primers shown in Table 1. Results from actin were used for normalization. cDNA was amplified in a ABI 7900HT Fast Real-Time PCR System (Life Technologies) in 20-µL reaction mixtures containing FastStart DNA Master SYBR Green I Mix reagent kit (Roche, Basel, Switzerland), using the standard qPCR conditions (40 cycles of 95°C for 15 s, followed by 60°C for 1 min) and dissociation curve in the control software of SDS 2.4 (Life Technologies). Statistical analyses of the qRT-PCR data were performed using Student's *t-*test (SPSS version 19).

### Nucleotide sequence accession number

The nucleotide sequences of the *dcl-1*, *dcl-2* and *qde-2* genes of *P. marneffei* have been deposited in GenBank under accession no. KC686608, KC686609 and KC686610 respectively. The Illumina small RNA sequences have been deposited in SRA NCBI database under accession no. SRX306604.

## Results

### Identification of *P. marneffei* small RNAs by deep sequencing

**Figure 2:**
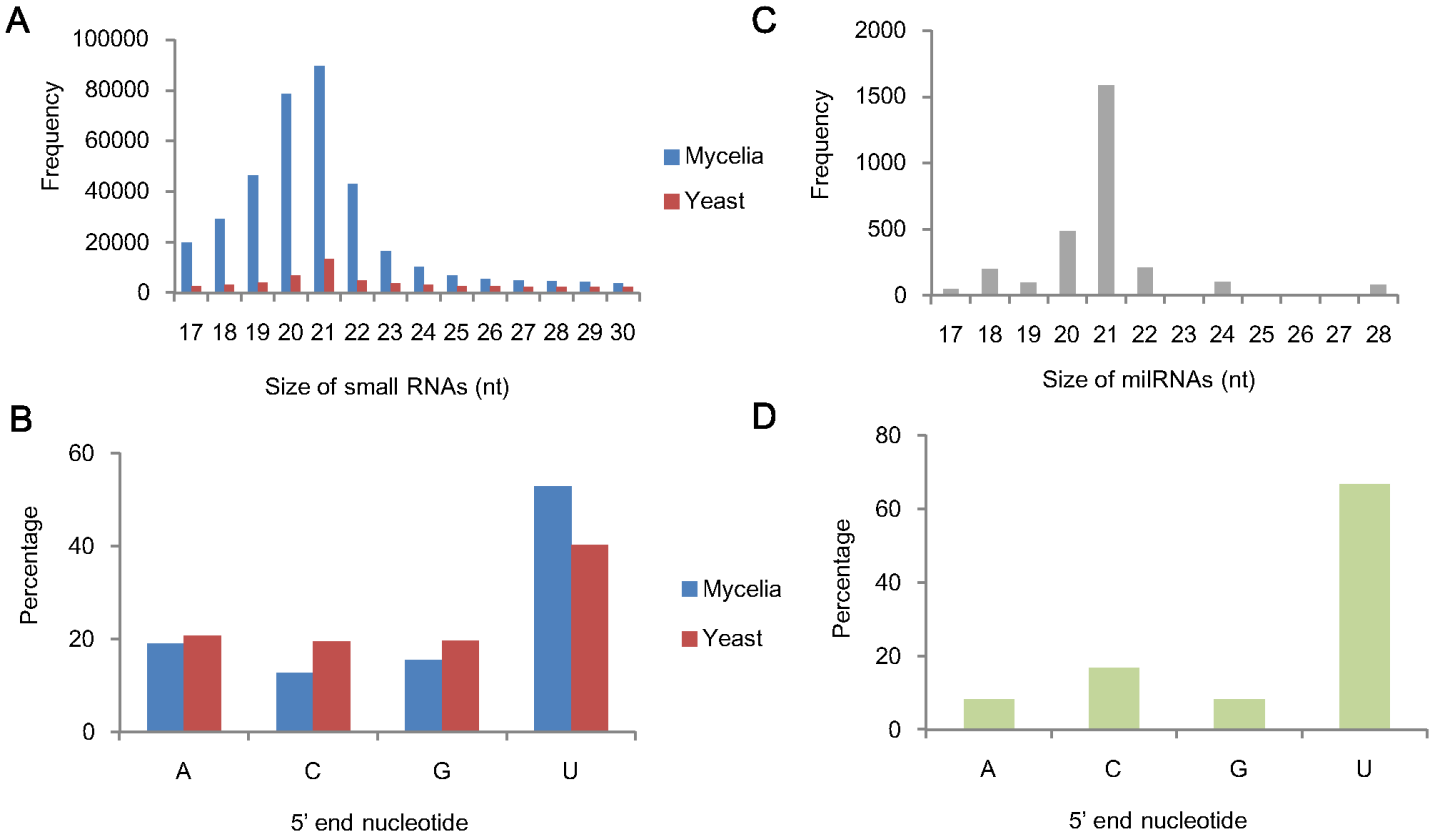
Characterization of small RNAs and milRNAs in *P. marneffei*. (A) Size distribution and (B) nucleotide frequency of the 5′ end of small RNAs in mycelial and yeast phase. (C) Size distribution and (D) nucleotide frequency of the 5′ end of the 24 milRNA candidates.

To examine small RNA species in the two growth phases of *P. marneffei*, cDNA libraries of small RNAs ≤60 nt extracted from mold and yeast cultures respectively were sequenced using the Illumina/Solexa Genome Analyzer IIx. The total number of both raw and filtered reads from mycelial and yeast phase was similar (Table 2). However, small RNAs were more abundant in mycelial than yeast phase of *P. marneffei*. We obtained a total of 3,155,063 and 270,782 high-quality, small RNA sequences of size 17–30 nt from mycelia and yeast phases respectively that perfectly match the *P. marneffei* genome. Among these, 362,805 and 56,543 unique small RNA sequences were identified from mycelial and yeast phases respectively (Fig. 2A). Most (89%) of the small RNAs identified from mycelial phase were 17–23 nt long, with the peak at 20–21 nt, and had a strong preference (52.82%) for 5′U (Fig. 2B), a known phenomenon in small RNAs of animals and plants [54], [55].

**Table 2:** Analysis of total and small RNA sequences in mycelial and yeast phase of *P. marneffei.*

	Total reads	Unique reads
Mycelial		
Raw reads	39,809,400	
Filtered reads	27,914,677	
Adaptors or rRNA reads	11,435,372	
Small RNA reads (17–30 nt)	6,910,710	1,077,964
Small RNA reads (17–30 nt) mapped to *P. marneffei* genome	3,155,063	362,805
Yeast		
Raw reads	36,999,600	
Filtered reads	28,424,899	
Adaptors or rRNA reads	15,670,222	
Small RNA reads (17–30 nt)	768,705	165,545
Small RNA reads (17–30 nt) mapped to *P. marneffei* genome	270,782	56,543

### Potential milRNAs in *P. marneffei*

Based on the distinguishing feature of known plant and animal miRNAs, 24 milRNA candidates, with flanking sequences forming hairpin secondary structures and at least five reads, were identified (Table 3). Their size distribution was shown in Fig. 2C, with a peak at 21 nt. There was also strong preference for U at their 5′ termini (67%, 16 of the 24 milRNA candidates) (Fig. 2D). These include 17 potential milRNAs (2,502 reads) in mycelial phase and seven potential milRNAs (232 reads) in yeast phase respectively (Table 3).

**Table 3:** Potential milRNA candidates in mycelial and yeast phase of *P. marneffei*.

milRNA	Sequence (5′–3′)	Length (nt)	Reads
*PM-milR-M1*	GAGAAACGCCUUAUGAUCGAC	21	1482
PM-milR-M1*	UGACUCGAAGAGCCUCUA	18	1
*PM-milR-M2*	GUCCUAUAGUAAAGCCAGUC	20	10
PM-milR-M2*	AUUUCUAGGCUAUAAAAGCUU	21	1
*PM-milR-MC3*	UGAUAUCAAAGUGGGCUAUC	20	351
*PM-milR-MC4*	UCAAGUCAACCCUUACUC	18	198
*PM-milR-MC5*	UUGCUAUGAUGAAAGCUGAGCA	22	127
*PM-milR-MC6*	AACGUUUAAAUUUCCGAUACAAUU	24	101
*PM-milR-MC7*	UAGGAUUAGGAUUAGGAUUA	20	97
*PM-milR-MC8*	UUUCUACAGCUGCUGAACGUC	21	44
*PM-milR-MC9*	UUGGCGUUGGGUGUAAUUG	19	22
*PM-milR-MC10*	UCGACUGGCUCACCUGAUGCC	21	14
*PM-milR-MC11*	UCGAUGUACUUCCUUGUGGA	20	12
*PM-milR-MC12*	UGUUCAUCGAUCUGCUGUAGA	21	9
*PM-milR-MC13*	UGCCACUCGAUCAUCUUGGG	20	8
*PM-milR-MC14*	UAAGAGCUGUACAUAUGUAAG	21	8
*PM-milR-MC15*	AUCCGGAUCGAGUUAUUCAC	20	8
*PM-milR-MC16*	CAUAAGGUCGAGAGUCUCGCA	21	6
*PM-milR-MC17*	UGGCGGACGCGAUGGUGGAGG	21	5
*PM-milR-YC1*	UGCCAUUGCUAAGUCAAGG	19	76
*PM-milR-YC2*	CAGCGGUGAUGACAACC	17	47
*PM-milR-YC3*	CCGCUUCUAAAAUUGCUAGAGC	22	44
*PM-milR-YC4*	UUGCUAUGAUGAAAGCUGAGCA	22	30
*PM-milR-YC5*	UUUCUUGUCUACCUUUCGAGU	21	19
*PM-milR-YC6*	UUCUCGGUGGCGAUGUCCAUU	21	8
*PM-milR-YC7*	CCUUCAGAUCUGGGCUAUGCCC	22	8

### Identification and sequence analysis of *dcl-1*, *dcl-2* and *qde-2* genes

**Figure 3:**
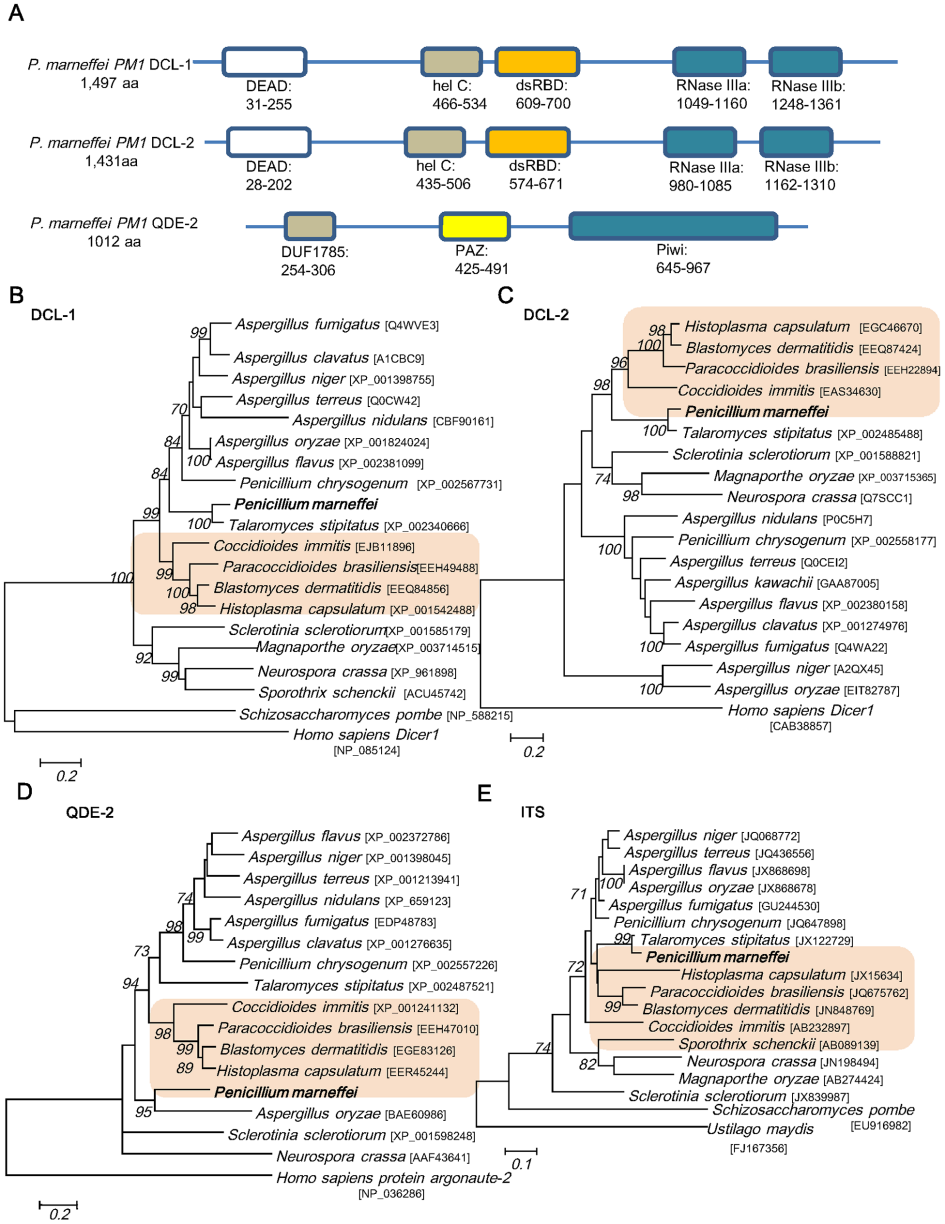
Sequence analysis of *dcl-1*, *dcl-2* and *qde-2* genes in *P. marneffei*. (A) Predicted domains of Dicer and QDE-2 proteins in *P. marneffei* strain PM1. Black bars represent the full protein sequence. The boxes represent the identified domains, each with its starting and stopping amino acid. Both DCL-1 and DCL-2 of *P. marneffei* contain a DEAD box, a helicase C domain (hel C), a double stranded RNA binding domain (dsRBD), and two RNase III domains (RNase IIIa and RNase IIIb). QDE-2 contains a PAZ domain ,a Piwi domain and a DUF1785 domains. Phylogenetic tree showing the relationship of predicted protein sequences of (B) *dcl-1*, (C) *dcl-2*, (D) *qde-2* and (E) ITS of *P. marneffei* to homologues in other fungi constructed by maximum-likelihood method with *Homo sapiens* (DCL-1,DCL-2 and QDE-2) and *Ustilago maydis* (ITS) as the root. The thermal dimorphic pathogenic fungi are highlighted. A total of 914, 764 and 525 amino acid positions for *dcl-1*, *dcl-2* and *qde-2* and 465 nucleotide positions for ITS were included in the analysis respectively. Bootstrap values were calculated as percentages from 1000 replicates and only values ≥70% were shown. The scale bars indicate the estimated number of substitutions per 5, 5, 5 amino acids and 10 bases respectively. Names and accession numbers are given as cited in GenBank database.

Using the respective homologues of *N. crassa* for BLAST search of *P. marneffei* strain *PM1* draft genome sequence, two *dcl* genes, *dcl-1* and *dcl-2*, encoding putative Dicer-like proteins and a gene, *qde-2*, encoding a putative Argonaute-like protein were identified (Fig. 3A). Dicer and Argonaute proteins are known to be involved in the biogenesis of miRNAs in animals and plants [56], [57], [58]. The *dcl-1* gene is 5,383 bp in length, comprising 15 introns with total length of 889 bp. The resultant mRNA encodes 1,497 amino acid residues with a predicted molecular mass of 170.31 kDa. The *dcl-2* gene is 4,636 bp in length, comprising six introns with total length of 340 bp. The resultant mRNA encodes 1,431 amino acids with a predicted mass of 161.15 kDa. These putative proteins possessed 42% and 32% amino acid identities to the DCL-1 and DCL-2 of *N. crassa* respectively. Both predicted proteins contain all four domains characteristic of the Dicer family. Two RNase III domains are present in the C-terminal region, and a DEAD-box ATP binding domain is present in the N-terminal region. In between there are RNA helicase and double stranded RNA binding domains. The *qde-2* gene is 3,199 bp in length, comprising three introns with total length of 160 bp. The resultant mRNA encodes 1,012 amino acid residues with a predicted molecular mass of 111.75 kDa. The predicted QDE-2 protein possessed 35% amino acid identity to the QDE-2 of *N. crassa*. It contains two characteristic domains of the argonaute family, PAZ and Piwi domains, and the DUF1785 domain conserved in many argonaute proteins. The domain organization of DCL-1, DCL-2 and QDE-2 of *P. marneffei* is similar to that of the corresponding homologues in *N. crassa* [59].

Our previous study based on mitochondrial genome sequence has shown that *P. marneffei* is phylogenetically more closely related to those of filamentous fungi, including *Aspergillus* species, than yeasts [34]. Phylogenetic analysis of both ITS, another important marker for fungal identification and phylogeny, and *dcl-1* gene showed that the corresponding sequences in *P. marneffei* were most closely related to *Talaromyces stipitatus* (a teleomorph of *Penicillium emmonsii*), *Penicillium chrysogenum* and *Aspergillus* spp. (Fig. 3). In contrast, phylogenetic analysis of *dcl-2* and *qde-2* genes showed a different evolutionary topology. The *dcl-2* of *P. marneffei* and its homologue in *T. stipitatus* are more closely related to those of the thermal dimorphic pathogenic fungi, *Histoplasma capsulatum*, *Blastomyces dermatitidis*, *Paracoccidioides brasiliensis* and *Coccidioides immitis* than to *P. chrysogenum* and *Aspergillus* spp., suggesting the co-evolution of *dcl-2* among the thermal dimorphic fungi. On the other hand, *qde-2* of *P. marneffei* is most closely related to its homologues in other thermal dimorphic fungi than to that in *T. stipitatus*, *P. chrysogenum* and *Aspergillus* spp.

### Differential mRNA expression of *dcl-1*, *dcl-2* and *qde-2* in mycelial and yeast phase

**Figure 4:**
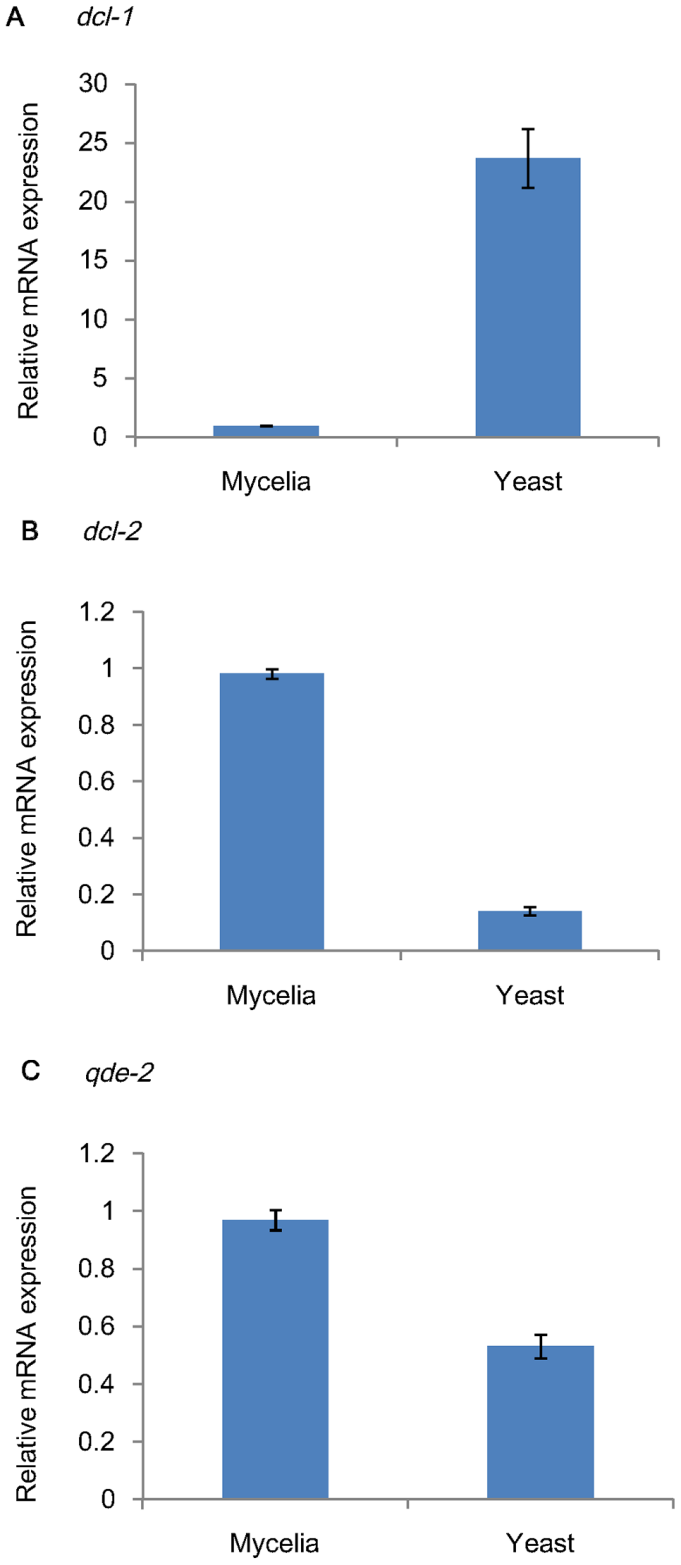
Relative mRNA expression of (A) *dcl-1*, (B) *dcl-2* and (C) *qde-2* genes in mycelial and yeast phase of *P. marneffei* by qRT-PCR. Results were obtained from five independent experimental replicates.

The mRNA expression level of *dcl-1* in yeast phase was significantly higher than mycelial phase by 25 folds (*P*<0.001 by student t test). In contrast, the mRNA expression levels of *dcl-2* and *qde-2* were higher in mycelial phase than in yeast phase by 7 folds and 2 folds respectively (*P*<0.001 by Student's t-test) (Fig. 4).

### Dicer-dependent biogenesis of milRNA in *P. marneffei*

**Figure 5:**
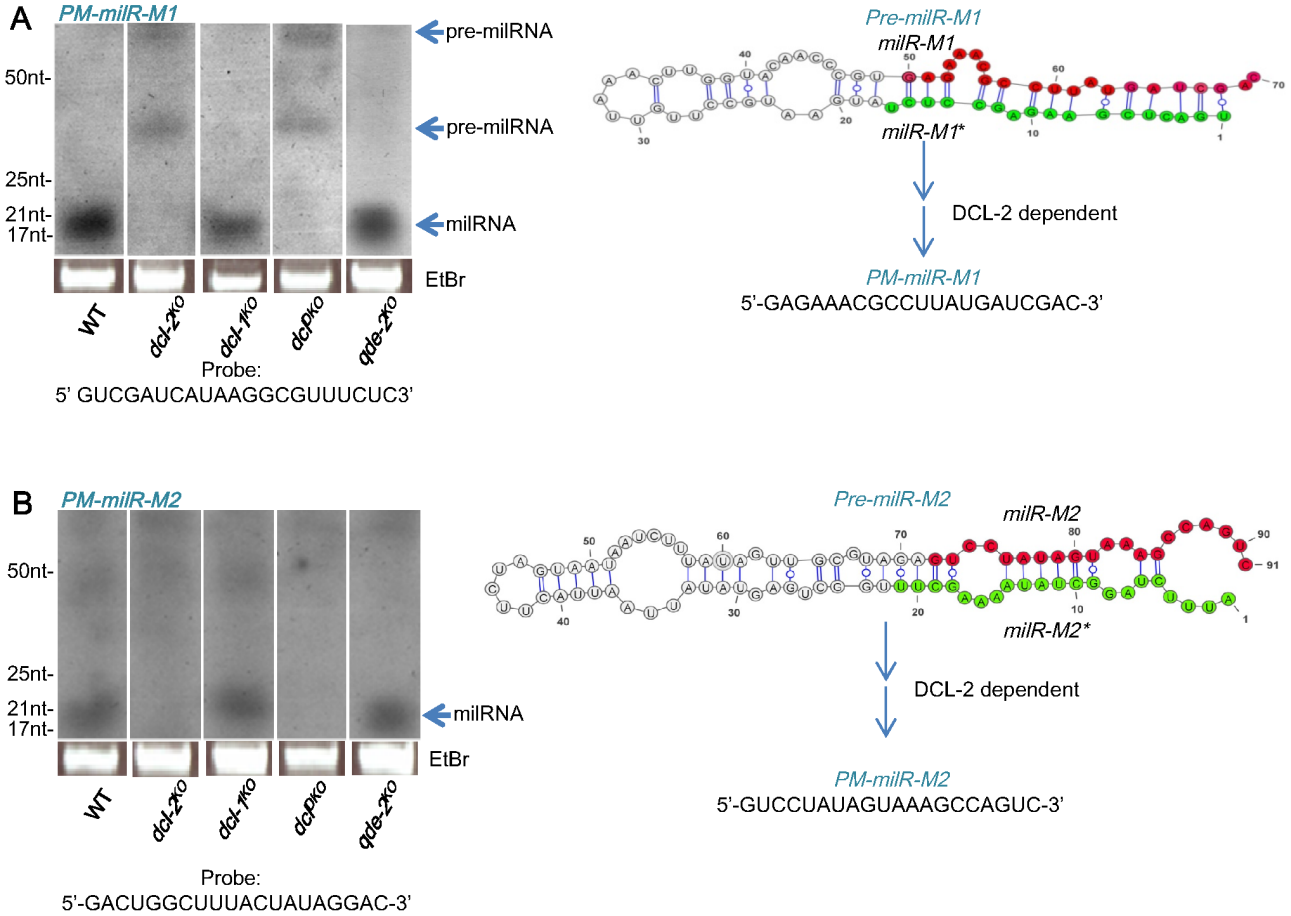
milRNA biogenesis mechanism for *PM-milR-M1* and *PM-milR-M2* in *P. marneffei*. Northern blot analyses of small RNA samples in wild-type (WT), *dcl-1^KO^*, *dcl-2^KO^*, *dcl^DKO^* and *qde-2^KO^* strains of *P. marneffei* showing that the production of milRNA of (A) *PM-milR-M1* and (B) *PM-milR-M2* requires DCL-2 but not DCL-1 or QDE-2. The ethidium bromide-stained denaturing gel in the bottom panel showed equal loading of RNA. Predicted structures of pre-milRNA of *PM-milR-M1* and *PM-milR-M2*, with their milRNA and paired milRNA* sequences as labeled in red and green respectively, are shown next to the northern blot analyses. The probe sequences used for northern blot analyses are marked.

Northern blot analyses showed the production of milRNAs from two of the predicted milRNA loci, *PM-milR-M1* and *PM-milR-M2*, both from mycelial phase of *P. marneffei*, with their predicted milRNA precursor (pre-milRNA) structures shown in Fig. 5. Their predicted precursors were approximately 70-nt and 91-nt in size and had negative folding free energies of −17.86 kcal mol^−1^ and -23.88 kcal mol^−1^ according to RNAfold (http://www.tbi.univie.ac.at/~ivo/RNA/RNAfold.html) for *PM-milR-M1* and *PM-milR-M2* respectively, comparable to those of known miRNA or milRNA precursors [31], [60], [61]. The majority of small RNA sequences of *PM-milR-M1* and *PM-milR-M2* correspond to one arm of the hairpin (the milRNA arm), with a total of 1482 small RNAs sequenced from *PM-milR-M1* and 10 small RNAs sequenced from *PM-milR-M2* (Table 3). In addition, small RNAs (milRNA^*^) matched to the complementary arm of the hairpin of *PM-milR-M1* and *PM-milR-M2* were also sequenced, but at much lower frequencies. In contrast to many small RNAs in which the miRNA arm possesses a 5′U position, the milRNA of both *PM-milR-M1* and *PM-milR-M2* have a 5′G position (Fig. 4). The existence of milRNA^*^ and the presence of a 2 nt 3′ overhang in these milRNA/milRNA^*^ pairs are strong evidence that they are produced from a Dicer-like enzyme (Fig. 5) [62]. Since loci which produce mature miRNAs and miRNA^*^ sequences are considered miRNA loci, the two loci are tentatively named as *P. marneffei milR-1* (*PM-milR-1*) and *PM-milR-2*. The locus, *PM-milR-1*, was situated within the coding region of a hypothetical protein, whereas *PM-milR-2* was situated in the opposite strand of a pogo transposable element within a repeat region in the *P. marneffei* genome. The other 22 loci were considered milRNA candidates (named *PM-milR-MC3*, *MC4…MC17* for milRNA candidates in mycelial phase and *PM-milR-YC1…YC7* for those in yeast phase). These novel milRNAs or milRNA candidates showed no sequence similarity to known miRNAs miRBase as of March 2013.

To study the expression profile of *PM-milR-M1* and *PM-milR-M2* in mycelial and yeast phases, Northern blot analyses of small RNAs were performed, which cross-validated the Illumina sequencing results indicating mycelial-specific expression (Fig. 5A) with little or no expression in yeast phase (data not shown) of wild-type strain PM1. To assess the role of *dcl-1*, *dcl-2* and *qde-2* in the biogenesis of *PM-milR-M1* and *PM-milR-M2*, *dcl-1^KO^*, *dcl-2^KO^*, *dcl^DKO^* and *qde-2^KO^* mutants were generated using homologous recombination. All deletion mutants exhibited similar growth rates and phenotypic characteristics to wild-type strain in both mycelial and yeast phase cultures, although the *dcl^DKO^* mutant exhibited poor sporulation and reduced red pigment production compared to wild-type strain upon transition from yeast to mycelial phase on sabouraud agar (data not shown).

Northern blot analysis of *PM-milR-M1* in wild-type and deletion mutants showed that a band corresponding to the mature milRNA product with approximate size of 21 nt was present in wild-type strain, *dcl-1^KO^* and *qde-2^KO^* mutants, but absent in *dcl-2^KO^* and *dcl^DKO^* mutants (Fig. 5A). Moreover, a band with approximate size of 70 nt, which matches the size of the predicted precursor of milRNA (pre-milRNA) of *PM-milR-M1*, was present in *dcl-2^KO^* and *dcl^DKO^* mutants but not in wild-type strain, *dcl-1^KO^* or *qde-2^KO^* mutants. In addition, a band of approximately 30 nt is also seen in *dcl-2^KO^* and *dcl^DKO^* mutants but not in wild-type strain, *dcl-1^KO^* or *qde-2^KO^* mutants, which may represent an intermediate product of the precursor. This suggested that DCL-2 protein is required for the biogenesis of mature milRNA from *PM-milR-M1* and that the band at about 70 nt is likely the pre-milRNA. In the *dcl-1^KO^* and *qde-2^KO^* mutants, the levels of mature milRNA were similar to that of wild-type, indicating that DCL-1 and QDE-2 are not required for milRNA production from *PM-milR-M1*. As for *PM-milR-M2*, the band corresponding to its mature milRNA product, with approximate size of 20 nt, was also present in wild-type strain, *dcl-1^KO^* and *qde-2^KO^* mutants, but was absent in *dcl-2^KO^* and *dcl^DKO^* mutants (Fig. 5A). This suggested that DCL-2 protein is also required for the biogenesis of mature milRNA from *PM-milR-M2*. In the *dcl-1^KO^* and *qde-2^KO^* mutants, the levels of mature milRNA were similar to that of wild-type, indicating that DCL-1 and QDE-2 are not required for milRNA production from *PM-milR-M2*.

### Predicted milRNA targets in *P. marneffei*

Among the 24 potential milRNA candidates identified in the present study, 21 were predicted to have potential targets while three have no predicted targets (Supplementary Table S1). One of the candidates, *PM-milR-MC17*, was predicted to have up to 353 potential targets. These milRNAs candidates with predicted targets bind either perfectly or imperfectly complementary sequences. However, both *PM-milR-M1* and *PM-milR-M2* were predicted to bind complementary sequences of their targets imperfectly, similar to miRNAs in animals and the filamentous fungus, *N. crassa* [30]. The predicted targets of *PM-milR-M1* include a putative Ran-binding protein RanBP10, a putative benzoate 4-monooxygenase cytochrome P450 and a conserved hypothetical protein. RanBP10 is a cytoplasmic guanine nucleotide exchange factor that modulates noncentrosomal microtubules involved in mitosis, while cytochrome P450 catalyses diverse reactions in fungal primary and secondary metabolism, and xenobiotic detoxification. As for *PM-milR-M2*, 20 potential targets were predicted, which include 13 transposon or transposable elements and seven conserved hypothetical proteins.

### Regulation of target gene expression by *PM-milR-M1*

**Figure 6:**
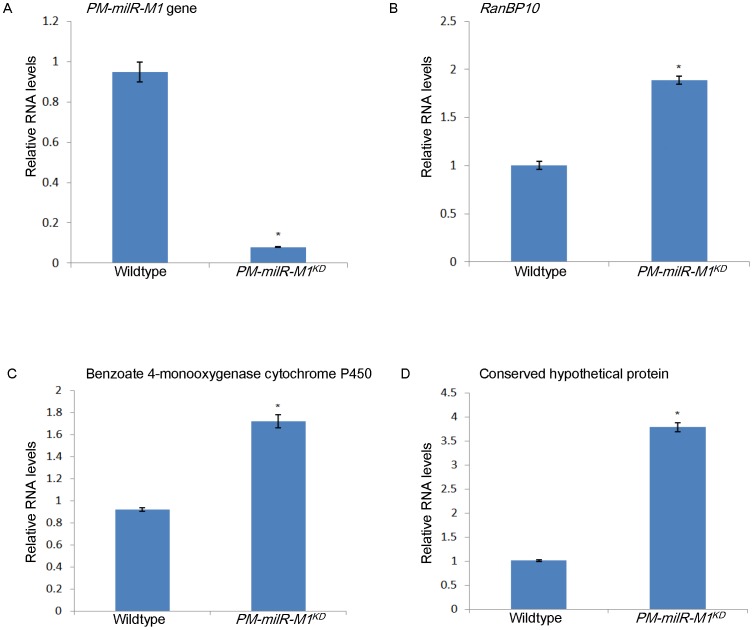
Regulation of target gene expression by *PM-milR-M1.* Relative mRNA expression of (A) *PM-milR-M1* gene, (B) *RanBP10*, (C) benzoate 4-monooxygenase cytochrome P450 and (D) a conserved hypothetical protein in mycelial phase of wild type strain PM1 and knockdown strain *PM-milR-M1^KD^* by qRT-PCR. Results were obtained from three independent experimental replicates.

To test for potential regulation of target gene expression by these milRNAs, we generated a knockdown strain of *PM-milR-M1* gene and measured the mRNA expression levels of the three predicted target genes. The knockdown strain, *PM-milR-M1^KD^*, only exhibited 8% transcription level of *PM-milR-M1* gene in mycelial phase compared to wild type strain PM1 (Fig. 6A). The mRNA expression levels of the three predicted targets, putative RanBP10, putative benzoate 4-monooxygenase cytochrome P450 and a conserved hypothetical protein, were upregulated in *PM-milR-M1^KD^* by 1.9 (Fig. 6B) , 1.7 (Fig. 6C) and 3.8 folds (Fig. 6D) respectively compared to wild type strain PM1 (*P*<0.05 by student t test).

## Discussion

This is the first report of milRNAs in a human thermal dimorphic pathogenic fungus and their differential expression in mycelial and yeast phases. RNAi proteins such as Dicer and Argonaute have been identified in many fungi, such as the model filamentous fungus *N. crassa* [63] and fission yeast *Schizosaccharomyces pombe* [64]. Although RNAi proteins were lost in the famous budding yeast *Saccharomyces cerevisiae*, the closely related species *Saccharomyces castelli* encoded a defected but functional Dicer-like homolog [65]. However, till 2005, no endogenous miRNAs have been reported in fungi but only reports of antisense transcripts encoded in the genome of *C. neoformans* [66]. No plant or animal-like miRNAs was found in *Aspergillus* species by computational analysis of six *Aspergillus* genomes (*Aspergillus nidulans*, *Aspergillus oryzae*, *Aspergillus fumigatus*, *Aspergillus terreus*, *Aspergillus clavatus*, and *Neosartorya fischeri*) [67]. It was therefore uncertain whether fungi have microRNAs until the recent discovery of milRNAs in the filamentous fungi, *N. crassa*, *S. sclerotiorum* and *M. anisopliae*, as well as the human pathogenic yeast, *C. neoformans* [30]–[33]. Nevertheless, the presence of milRNAs in human pathogenic filamentous and dimorphic fungi was largely unknown. We have previously shown that target gene expression can be specifically knocked down by an RNAi-based method in *P. marneffei* [36], [40]. Moreover, we found that two *dcl* genes encoding putative dicer-like proteins and a *qde-2* gene encoding a putative Argonaute-like protein, QDE-2, can be identified in *P. marneffei* strain PM1 draft genome, which are known to play key roles in the biogenesis of miRNAs and siRNAs [68]. Since miRNAs are important gene regulatory molecules in multicellular organisms, we hypothesized that *P. marneffei* possesses functional RNAi machinery and may encode miRNAs, which may be involved in the regulation of thermal dimorphism. In this study, using high throughput sequencing of small RNAs extracted from mycelial and yeast cultures of *P. marneffei*, we showed that small RNAs are more abundantly expressed in mycelial than yeast phase by >10 folds. The sequencing result is also in line with the more abundant small RNAs (approximately 20–24 nt) observed in mycelial than yeast phase upon Sybr Gold stained 12% denaturing polyacylamide gel electrophoresis (data not shown). After exclusion of other non-coding RNAs, a total of 2,734 reads were identified as potential milRNA candidates including 17 candidates in mycelial phase and seven in yeast phase, suggesting that milRNAs are differentially expressed in the two growth phases and may be more abundant in mycelial than yeast phase of *P. marneffei*. Two milRNAs, *PM-milR-M1* and *PM-milR-M2*, both expressed in mycelial phase, were confirmed by Northern blot analyses. They share similar characteristics to miRNAs in animals and plants, being dependent on a Dicer-like protein for production and arisen from highly specific stem-loop RNA precursors. *PM-milR-M1* was also shown to regulate the mRNA expression of its predicted target genes. The present results supported that dimorphic fungi may encode milRNAs which are likely conserved regulators of gene expression in diverse eukaryotes including fungi [18].

DCL-2 is likely a conserved protein involved in milRNA biogenesis among thermal dimorphic fungi. Dicer is a member of RNAse III family of nucleases and is responsible for miRNA processing in animals and plants [18]. While dicer-like proteins are known to be important for RNAi silencing in various fungi [36], [40], [69], [70], its role in milRNAs in fungi has been less well studied. A recent study on *N. crassa* has revealed diverse pathways in the generation of milRNAs and Dicer-independent small interfering RNAs (disiRNAs) [30]. In this study, the production of *PM-milR-M1* and *PM-milR-M2*, as well as the pre-milRNA of *PM-milR-M1*, was dependent on the presence of DCL-2 but not DCL-1 or QDE-2 in *P. marneffei*. The pre-milRNA of *PM-milR-M2* was not obvious upon Northern blot analyses, which may be due to degradation into small RNAs because of instability. No identifiable homologues of *PM-milR-M1* and *PM–milR-M2* could be in animals and plants, which supported the independent evolution of milRNAs in fungi [30], [33]. On the other hand, homologues of their precursors can be identified in *T. stipitatus* (data not shown). Nevertheless, it remains to be determined if such milRNA homologues are also expressed and processed in the same way. Interestingly, in contrast to ITS and *dcl-1* sequences which were both phylogenetically most closely related to the homologues in *T. stipitatus*, *P. chrysogenum* and *Aspergillus* spp., the *dcl-2* gene of *P. marneffei* is more closely related to the homologues in other geographically restricted thermal dimorphic fungi than to *P. chrysogenum* and *Aspergillus* spp.. This suggested that the *dcl-2* gene may have co-evolved among the thermal dimorphic fungi and serve similar function. Since these thermal dimorphic fungi are different from other fungi by their ability to cause systemic mycosis as intracellular yeasts and survive in natural environments as molds, it would be interesting to explore the potential role of DCL-2 in fungal dimorphism as well as virulence. In *N. crassa*, at least four different mechanisms that involved a combination of factors were identified for the production of milRNAs. In fact, apart from dicers and QDE-2, homologues of QDE-2 interacting protein (QIP) and mitochondrial ribosomal protein L3 (MRPL3), which were also involved in biogenesis of some milRNAs in *N. crassa* [30], can also be found in the *P. marneffei* genome, with 27–49% amino acid identities (data not shown). Further studies are required to explore for possible role of these proteins in milRNA biogenesis in *P. marneffei.*

In contrast to miRNAs from animals and plants which are known to play different functions from multicellular development to stress response, the potential function(s) of milRNAs in fungi remain to be determined. Some miRNAs in plants and animals are known to exhibit temporal or tissue-specific expression patterns [18], [71], [72]. As for fungi, a recent study showed that some milRNAs are differentially expressed in sclerotial development of *S. sclerotiorum* [31]. In *C. neoformans*, milRNAs were shown to cause transgene silencing via the canonical RNAi pathway and proposed to be play a role in regulating transposons and pseudogene expression [33]. In this study, we showed the mRNA expression level of *dcl-2* was higher in mycelial than yeast phase, suggesting that DCL-2 may function predominantly in the mycelial phase. This, in turn, may explain why *PM-milR-M1* and *PM–milR-M2* were only expressed in mycelial but not yeast form of *P. marneffei*. Therefore, it is likely that *PM-milR-M1* and *PM-milR-M2* are only produced from DCL-2 and serve important function during mycelial phase. A number of potential targets were predicted for both *PM-milR-M1* and *PM-milR-M2*. For example, the predicted targets of *PM-milR-M1* include *RanBP*10 and cytochrome P450, while transposon or transposable elements were the predominant predicted targets of *PM-milR-M2*. The targets of *PM-milR-M1* were also confirmed to be upregulated at the RNA level in the knockdown strain, *PM-milR-M1^KD^*, supporting the mRNA cleavage function of milRNAs. These results suggested that milRNAs in *P. marneffei* may regulate cell division, metabolism as well as transposons, although further studies are required to investigate their biological function. Nevertheless, the present study demonstrated the potential role of differential post-transcriptional control in different growth phases of thermal dimorphic fungi, which may provide new insights into the mechanism governing thermal dimorphism.

## Supporting Information

Table S1 Predicted targets of milRNAs in *P. marneffei*.
(DOCX)
