# Supplementary material for: Identification of MicroRNA-Like RNAs in Mycelial and Yeast Phases of the Thermal Dimorphic Fungus Penicillium marneffei
Source: PLoS Negl Trop Dis. 2013 Aug 22;7(8):e2398. doi: 10.1371/journal.pntd.0002398 (PMC3749987; doi:10.1371/journal.pntd.0002398)
Supplement: Table S1 — Predicted targets of milRNAs in P. marneffei. (DOCX) [file pntd.0002398.s001.docx]

Supplementary Table S1. Predicted targets of milRNAs in *P. marneffei*

| milRNAs | target mRNA | site position | target length | Remarks: |
| --- | --- | --- | --- | --- |
| *PM-milR-M1* | XM_002153120.1\| Ran-binding protein (RanBP10), putative, mRNA | 1385 | 2334 |  |
| *PM-milR-M1* | XM_002153301.1\| conserved hypothetical protein, mRNA | 4470 | 4731 |  |
| *PM-milR-M1* | XM_002146036.1\| benzoate 4-monooxygenase cytochrome P450, putative, mRNA | 267 | 1200 |  |
| *PM-milR-M2* | XM_002144076.1\| transposon, putative, mRNA | 536 | 1757 |  |
| *PM-milR-M2* | XM_002144493.1\| conserved hypothetical protein, mRNA | 365 | 1203 |  |
| *PM-milR-M2* | XM_002146057.1\| pogo transposable element, putative, mRNA | 518 | 1821 |  |
| *PM-milR-M2* | XM_002146378.1\| pogo transposable element, putative, mRNA | 227 | 1314 |  |
| *PM-milR-M2* | XM_002146381.1\| pogo transposable element, putative, mRNA | 548 | 1698 |  |
| *PM-milR-M2* | XM_002146475.1\| pogo transposable element, putative, mRNA | 518 | 1691 |  |
| *PM-milR-M2* | XM_002147154.1\| conserved hypothetical protein, mRNA | 444 | 1531 |  |
| *PM-milR-M2* | XM_002148112.1\| transposon, putative, mRNA | 518 | 1521 |  |
| *PM-milR-M2* | XM_002148442.1\| conserved hypothetical protein, mRNA | 227 | 1245 |  |
| *PM-milR-M2* | XM_002148795.1\| transposon, putative, mRNA | 518 | 1668 |  |
| *PM-milR-M2* | XM_002149109.1\| conserved hypothetical protein, mRNA | 227 | 756 |  |
| *PM-milR-M2* | XM_002149110.1\| pogo transposable element, putative, mRNA | 518 | 1668 |  |
| *PM-milR-M2* | XM_002149111.1\| conserved hypothetical protein, mRNA | 518 | 1281 |  |
| *PM-milR-M2* | XM_002150101.1\| transposon, putative, mRNA | 569 | 1233 |  |
| *PM-milR-M2* | XM_002150513.1\| pogo transposable element, putative, mRNA | 518 | 1852 |  |
| *PM-milR-M2* | XM_002150848.1\| transposon, putative, mRNA | 518 | 1302 |  |
| *PM-milR-M2* | XM_002150952.1\| conserved hypothetical protein, mRNA | 478 | 710 |  |
| *PM-milR-M2* | XM_002151776.1\| pogo transposable element, putative, mRNA | 518 | 1560 |  |
| *PM-milR-M2* | XM_002151777.1\| conserved hypothetical protein, mRNA | 521 | 1623 |  |
| *PM-milR-M2* | XM_002153191.1\| pogo transposable element, putative, mRNA | 401 | 1557 |  |
| *PM-milR-MC3* | XM_002152788.1\| conserved hypothetical protein, mRNA | 1852 | 5236 | perfectly complementary |
| *PM-milR-MC3* | XM_002149766.1\| conserved hypothetical protein, mRNA | 1641 | 4431 | perfectly complementary |
| *PM-milR-MC3* | XM_002148515.1\| conserved hypothetical protein, mRNA | 1572 | 4494 | perfectly complementary |
| *PM-milR-MC3* | XM_002147049.1\| conserved hypothetical protein, mRNA | 1641 | 4671 | perfectly complementary |
| *PM-milR-MC3* | XM_002147049.1\| conserved hypothetical protein, mRNA | 1640 | 4671 |  |
| *PM-milR-MC3* | XM_002148515.1\| conserved hypothetical protein, mRNA | 1571 | 4494 |  |
| *PM-milR-MC3* | XM_002149766.1\| conserved hypothetical protein, mRNA | 1640 | 4431 |  |
| *PM-milR-MC3* | XM_002151360.1\| conserved hypothetical protein, mRNA | 1016 | 1429 |  |
| *PM-milR-MC3* | XM_002152788.1\| conserved hypothetical protein, mRNA | 1851 | 5236 |  |
| *PM-milR-MC4* | no predicted targets |  |  |  |
| *PM-milR-MC5* | XM_002143303.1\| hypothetical protein, mRNA | 856 | 956 |  |
| *PM-milR-MC5* | XM_002149698.1\| protein kinase, putative, mRNA | 730 | 3773 |  |
| *PM-milR-MC6* | no predicted targets |  |  |  |
| *PM-milR-MC7* | XM_002144095.1\| conserved hypothetical protein, mRNA | 1101 | 1421 |  |
| *PM-milR-MC7* | XM_002145998.1\| CDP-diacylglycerol-inositol 3-phosphatidyltransferase PIS, mRNA | 13 | 1733 |  |
| *PM-milR-MC7* | XM_002146689.1\| pyroglutamyl peptidase type I, putative, mRNA | 811 | 1356 |  |
| *PM-milR-MC8* | XM_002143560.1\| ubiquitin conjugating enzyme, putative, mRNA | 3383 | 3554 |  |
| *PM-milR-MC8* | XM_002145926.1\| hypothetical protein, mRNA | 165 | 1422 |  |
| *PM-milR-MC8* | XM_002147636.1\| conserved hypothetical protein, mRNA | 1105 | 4522 |  |
| *PM-milR-MC8* | XM_002147898.1\| conserved hypothetical protein, mRNA | 1302 | 2949 |  |
| *PM-milR-MC8* | XM_002150344.1\| tryptophanyl-tRNA synthetase, mRNA | 1137 | 1748 |  |
| *PM-milR-MC8* | XM_002150345.1\| tryptophanyl-tRNA synthetase, mRNA | 1192 | 1803 |  |
| *PM-milR-MC8* | XM_002153352.1\| short-chain dehydrogenase/reductase, putative, mRNA | 833 | 1023 |  |
| *PM-milR-MC9* | XM_002147966.1\| 5-proFAR isomerase His6, putative, mRNA | 1208 | 1596 |  |
| *PM-milR-MC9* | XM_002143711.1\| DUF221 domain protein, putative, mRNA | 1223 | 3060 |  |
| *PM-milR-MC9* | XM_002144412.1\| dDENN domain protein, mRNA | 477 | 4125 |  |
| *PM-milR-MC9* | XM_002145846.1\| acid sphingomyelinase, putative, mRNA | 644 | 1884 |  |
| *PM-milR-MC9* | XM_002147966.1\| 5-proFAR isomerase His6, putative, mRNA | 1207 | 1596 | perfectly complementary |
| *PM-milR-MC9* | XM_002150722.1\| isopropanol dehydrogenase, putative, mRNA | 215 | 1116 |  |
| *PM-milR-MC9* | XM_002151377.1\| nuclear pore complex protein Nup107, putative, mRNA | 14 | 3207 |  |
| *PM-milR-MC10* | XM_002144658.1\| GPI transamidase component Gpi16, putative, mRNA | 160 | 1952 |  |
| *PM-milR-MC10* | XM_002145491.1\| nuclear cohesin complex subunit (Psc3), putative, mRNA | 2017 | 4206 |  |
| *PM-milR-MC10* | XM_002145572.1\| hypothetical protein, mRNA | 1098 | 1131 |  |
| *PM-milR-MC10* | XM_002145754.1\| conserved hypothetical protein, mRNA | 428 | 1081 |  |
| *PM-milR-MC10* | XM_002146417.1\| L-ornithine N5-oxygenase SidA, mRNA | 368 | 2084 |  |
| *PM-milR-MC10* | XM_002146889.1\| DNA replication initiation factor Cdc45, mRNA | 270 | 3001 |  |
| *PM-milR-MC10* | XM_002148504.1\| NF-X1 finger transcription factor, putative, mRNA | 414 | 3546 |  |
| *PM-milR-MC10* | XM_002148702.1\| conserved hypothetical protein, mRNA | 996 | 1418 |  |
| *PM-milR-MC10* | XM_002148794.1\| urease accessory protein UreG, putative, mRNA | 353 | 1232 |  |
| *PM-milR-MC10* | XM_002150625.1\| amino acid permease, putative, mRNA | 1534 | 1915 |  |
| *PM-milR-MC10* | XM_002152780.1\| acetyltransferase, GNAT family, putative, mRNA | 324 | 919 |  |
| *PM-milR-MC10* | XM_002152970.1\| transcription factor (Sin3), putative, mRNA | 3414 | 4785 |  |
| *PM-milR-MC10* | XM_002153528.1\| conserved hypothetical protein, mRNA | 2775 | 3763 |  |
| *PM-milR-MC10* | XM_002143182.1\| conserved hypothetical protein, mRNA | 88 | 1700 |  |
| *PM-milR-MC10* | XM_002144551.1\| conserved hypothetical protein, mRNA | 1203 | 2472 |  |
| *PM-milR-MC10* | XM_002145021.1\| phosphotidylinositol kinase Tel1, putative, mRNA | 3552 | 8765 |  |
| *PM-milR-MC10* | XM_002145353.1\| C6 transcription factor, putative, mRNA | 489 | 2682 |  |
| *PM-milR-MC10* | XM_002145900.1\| conserved hypothetical protein, mRNA | 1730 | 2289 |  |
| *PM-milR-MC10* | XM_002147805.1\| UBA/TS-N domain protein, mRNA | 766 | 2833 |  |
| *PM-milR-MC10* | XM_002148424.1\| aldehyde dehydrogenase, putative, mRNA | 238 | 1410 |  |
| *PM-milR-MC10* | XM_002149731.1\| fatty acid synthase subunit beta, putative, mRNA | 3654 | 6138 |  |
| *PM-milR-MC10* | XM_002150328.1\| translation factor pelota, putative, mRNA | 1049 | 1302 |  |
| *PM-milR-MC10* | XM_002151211.1\| SET domain protein, mRNA | 1293 | 2246 |  |
| *PM-milR-MC10* | XM_002152220.1\| monocarboxylate permease, putative, mRNA | 269 | 1949 |  |
| *PM-milR-MC10* | XM_002152640.1\| oxysterol binding protein (Orp8), putative, mRNA | 249 | 2010 |  |
| *PM-milR-MC10* | XM_002152641.1\| oxysterol binding protein (Orp8), putative, mRNA | 249 | 2406 |  |
| *PM-milR-MC10* | XM_002152832.1\| conserved hypothetical protein, mRNA | 81 | 1640 |  |
| *PM-milR-MC10* | XM_002153112.1\| conserved hypothetical protein, mRNA | 1037 | 1602 |  |
| *PM-milR-MC11* | XM_002151533.1\| phosphoglycerate kinase PgkA, putative, mRNA | 1821 | 2072 |  |
| *PM-milR-MC11* | XM_002143366.1\| conserved hypothetical protein, mRNA | 539 | 954 |  |
| *PM-milR-MC11* | XM_002143532.1\| conserved hypothetical protein, mRNA | 1685 | 2076 |  |
| *PM-milR-MC11* | XM_002144993.1\| ATP dependent RNA helicase (Dbp7), putative, mRNA | 290 | 2440 |  |
| *PM-milR-MC11* | XM_002145208.1\| conserved hypothetical protein, mRNA | 2273 | 2502 |  |
| *PM-milR-MC11* | XM_002145845.1\| 6-phosphogluconate dehydrogenase, decarboxylating, mRNA | 227 | 1814 |  |
| *PM-milR-MC11* | XM_002148867.1\| conserved hypothetical protein, mRNA | 109 | 2589 |  |
| *PM-milR-MC11* | XM_002150791.1\| nonribosomal peptide synthase, putative, mRNA | 21388 | 23706 |  |
| *PM-milR-MC11* | XM_002151533.1\| phosphoglycerate kinase PgkA, putative, mRNA | 1820 | 2072 | perfectly complementary |
| *PM-milR-MC12* | XM_002143267.1\| conserved hypothetical protein, mRNA | 544 | 1131 |  |
| *PM-milR-MC12* | XM_002144529.1\| actin cortical patch component, putative, mRNA | 756 | 2062 |  |
| *PM-milR-MC12* | XM_002143357.1\| MFS sugar transporter, putative, mRNA | 1560 | 1785 |  |
| *PM-milR-MC12* | XM_002149005.1\| conserved hypothetical protein, mRNA | 1220 | 1281 |  |
| *PM-milR-MC12* | XM_002151349.1\| alkaline dihydroceramidase Ydc1, putative, mRNA | 499 | 1809 |  |
| *PM-milR-MC12* | XM_002152758.1\| hypothetical protein, mRNA | 467 | 900 |  |
| *PM-milR-MC12* | XM_002153341.1\| tubulin-specific chaperone c, putative, mRNA | 914 | 1407 |  |
| *PM-milR-MC13* | XM_002143573.1\| short-chain dehydrogenase/reductase family protein, putative, mRNA | 394 | 1131 |  |
| *PM-milR-MC13* | XM_002146227.1\| gamma-tubulin complex component GCP6, putative, mRNA | 1911 | 2994 |  |
| *PM-milR-MC13* | XM_002148722.1\| F-box and WD repeat-containing protein, mRNA | 334 | 2467 |  |
| *PM-milR-MC13* | XM_002150686.1\| conserved hypothetical protein, mRNA | 431 | 660 |  |
| *PM-milR-MC13* | XM_002144119.1\| quinone oxidoreductase, putative, mRNA | 837 | 1120 |  |
| *PM-milR-MC13* | XM_002146034.1\| conserved hypothetical protein, mRNA | 673 | 1540 |  |
| *PM-milR-MC13* | XM_002146249.1\| RNA-binding La domain protein, mRNA | 914 | 1389 |  |
| *PM-milR-MC13* | XM_002146539.1\| conserved hypothetical protein, mRNA | 774 | 1197 |  |
| *PM-milR-MC13* | XM_002148831.1\| structural maintenance of chromosome complex subunit SmcA, mRNA | 759 | 3566 |  |
| *PM-milR-MC13* | XM_002149451.1\| short-chain dehydrogenase, putative, mRNA | 355 | 937 |  |
| *PM-milR-MC13* | XM_002150156.1\| small monomeric GTPase SarA, putative, mRNA | 456 | 1266 |  |
| *PM-milR-MC13* | XM_002150157.1\| small monomeric GTPase SarA, putative, mRNA | 456 | 1323 |  |
| *PM-milR-MC13* | XM_002150325.1\| hypothetical protein, mRNA | 1883 | 3123 |  |
| *PM-milR-MC13* | XM_002153241.1\| cytokinesis protein SepA/Bni1, mRNA | 748 | 5349 |  |
| *PM-milR-MC14* | no predicted targets |  |  |  |
| *PM-milR-MC15* | XM_002147735.1\| conserved hypothetical protein, mRNA | 208 | 1442 |  |
| *PM-milR-MC15* | XM_002150342.1\| conserved hypothetical protein, mRNA | 1008 | 3249 |  |
| *PM-milR-MC16* | XM_002144804.1\| DNA ligase, putative, mRNA | 1540 | 3018 |  |
| *PM-milR-MC16* | XM_002147153.1\| conserved hypothetical protein, mRNA | 686 | 1596 |  |
| *PM-milR-MC16* | XM_002145202.1\| fermentation associated protein (Csf1), putative, mRNA | 5247 | 9806 |  |
| *PM-milR-MC16* | XM_002149691.1\| conserved hypothetical protein, mRNA | 260 | 1392 |  |
| *PM-milR-MC16* | XM_002150141.1\| 60S ribosomal protein P0, mRNA | 245 | 1160 |  |
| *PM-milR-MC16* | XM_002153508.1\| conserved hypothetical protein, mRNA | 1290 | 1708 |  |
| *PM-milR-MC17* | XM_002143166.1\| conserved hypothetical protein, mRNA | 547 | 2290 |  |
| *PM-milR-MC17* | XM_002143188.1\| UTP-glucose-1-phosphate uridylyltransferase Ugp1, putative, mRNA | 613 | 1982 |  |
| *PM-milR-MC17* | XM_002143189.1\| UTP-glucose-1-phosphate uridylyltransferase Ugp1, putative, mRNA | 719 | 2088 |  |
| *PM-milR-MC17* | XM_002143251.1\| gibberellin 20 oxidase, putative, mRNA | 73 | 948 |  |
| *PM-milR-MC17* | XM_002143300.1\| chitin synthase activator (Chs3), putative, mRNA | 24 | 2925 |  |
| *PM-milR-MC17* | XM_002143308.1\| beta-glucosidase, putative, mRNA | 450 | 3051 |  |
| *PM-milR-MC17* | XM_002143637.1\| stress response protein Nst1, putative, mRNA | 847 | 4314 |  |
| *PM-milR-MC17* | XM_002143694.1\| malate synthase AcuE, mRNA | 1495 | 1880 |  |
| *PM-milR-MC17* | XM_002143697.1\| 5',5'''-P-1,P-4-tetraphosphate phosphorylase, putative, mRNA | 928 | 1409 |  |
| *PM-milR-MC17* | XM_002143913.1\| ubiquitin conjugating enzyme (UbcB), putative, mRNA | 357 | 969 |  |
| *PM-milR-MC17* | XM_002143926.1\| ABC multidrug transporter, putative, mRNA | 86 | 4392 |  |
| *PM-milR-MC17* | XM_002144039.1\| MFS sugar transporter, putative, mRNA | 781 | 1746 |  |
| *PM-milR-MC17* | XM_002144167.1\| GPI anchored serine-threonine rich protein, mRNA | 528 | 761 |  |
| *PM-milR-MC17* | XM_002144293.1\| C2H2 transcription factor Crz1, putative, mRNA | 1386 | 2480 |  |
| *PM-milR-MC17* | XM_002144434.1\| conserved hypothetical protein, mRNA | 7 | 1633 |  |
| *PM-milR-MC17* | XM_002144588.1\| conserved hypothetical protein, mRNA | 3304 | 3870 |  |
| *PM-milR-MC17* | XM_002144714.1\| cysteine-binding protein FliY, putative, mRNA | 1387 | 1878 |  |
| *PM-milR-MC17* | XM_002144759.1\| RNA helicase/RNAse III, putative, mRNA | 2685 | 4701 |  |
| *PM-milR-MC17* | XM_002144806.1\| conserved hypothetical protein, mRNA | 1125 | 1755 |  |
| *PM-milR-MC17* | XM_002144862.1\| hypothetical protein, mRNA | 42 | 888 |  |
| *PM-milR-MC17* | XM_002144899.1\| mRNA cap methyltransferase, mRNA | 688 | 2103 |  |
| *PM-milR-MC17* | XM_002145060.1\| conserved hypothetical protein, mRNA | 469 | 1801 |  |
| *PM-milR-MC17* | XM_002145201.1\| translation elongation factor EF-2 subunit, putative, mRNA | 537 | 2855 |  |
| *PM-milR-MC17* | XM_002145286.1\| kinesin family protein, mRNA | 4760 | 5689 |  |
| *PM-milR-MC17* | XM_002145338.1\| MFS transporter, putative, mRNA | 1139 | 2071 |  |
| *PM-milR-MC17* | XM_002145353.1\| C6 transcription factor, putative, mRNA | 1034 | 2682 |  |
| *PM-milR-MC17* | XM_002145384.1\| conserved hypothetical protein, mRNA | 2389 | 2828 |  |
| *PM-milR-MC17* | XM_002145457.1\| Rho guanyl nucleotide exchange factor, putative, mRNA | 3854 | 5908 |  |
| *PM-milR-MC17* | XM_002145475.1\| tetracycline-efflux transporter, putative, mRNA | 1677 | 1939 |  |
| *PM-milR-MC17* | XM_002145478.1\| PAP/25A associated domain family, mRNA | 1671 | 3180 |  |
| *PM-milR-MC17* | XM_002145525.1\| mitochondrial carrier protein (Leu5), putative, mRNA | 1230 | 1945 |  |
| *PM-milR-MC17* | XM_002145795.1\| conserved hypothetical protein, mRNA | 2871 | 4097 |  |
| *PM-milR-MC17* | XM_002145805.1\| ribosomal protein S13p/S18e, mRNA | 423 | 783 |  |
| *PM-milR-MC17* | XM_002145806.1\| ribosomal protein S13p/S18e, mRNA | 535 | 895 |  |
| *PM-milR-MC17* | XM_002145831.1\| RanGTP-binding protein, mRNA | 1191 | 1998 |  |
| *PM-milR-MC17* | XM_002145952.1\| cation diffusion facilitator 1, mRNA | 850 | 1496 |  |
| *PM-milR-MC17* | XM_002145963.1\| poly(A)-binding protein-dependent poly(A) ribonuclease, putative, mRNA | 1883 | 1966 |  |
| *PM-milR-MC17* | XM_002145964.1\| poly(A)-binding protein-dependent poly(A) ribonuclease, putative, mRNA | 130 | 2676 |  |
| *PM-milR-MC17* | XM_002146014.1\| conserved hypothetical protein, mRNA | 492 | 1614 |  |
| *PM-milR-MC17* | XM_002146259.1\| sexual development activator VeA, mRNA | 981 | 2092 |  |
| *PM-milR-MC17* | XM_002146301.1\| topoisomerase family protein TRF4, putative, mRNA | 1834 | 2336 |  |
| *PM-milR-MC17* | XM_002146362.1\| DNA excision repair protein Rad16, putative, mRNA | 609 | 3267 |  |
| *PM-milR-MC17* | XM_002146577.1\| RNA binding protein Jsn1, putative, mRNA | 504 | 3989 |  |
| *PM-milR-MC17* | XM_002146594.1\| NADH-ubiquinone oxidoreductase 64 kDa subunit, putative, mRNA | 254 | 2085 |  |
| *PM-milR-MC17* | XM_002146605.1\| short-chain dehydrogenase, putative, mRNA | 372 | 1689 |  |
| *PM-milR-MC17* | XM_002146662.1\| conserved hypothetical protein, mRNA | 175 | 3148 |  |
| *PM-milR-MC17* | XM_002146680.1\| RNP domain protein, mRNA | 39 | 1116 |  |
| *PM-milR-MC17* | XM_002146725.1\| retrograde regulation protein 2, mRNA | 954 | 1749 |  |
| *PM-milR-MC17* | XM_002146727.1\| thiazole biosynthesis enzyme, mRNA | 71 | 2558 |  |
| *PM-milR-MC17* | XM_002146773.1\| topisomerase II associated protein (Pat1), putative, mRNA | 798 | 2774 |  |
| *PM-milR-MC17* | XM_002146782.1\| C6 transcription factor, putative, mRNA | 1759 | 3353 |  |
| *PM-milR-MC17* | XM_002146815.1\| aldo-keto reductase (AKR13), putative, mRNA | 475 | 1032 |  |
| *PM-milR-MC17* | XM_002146960.1\| conserved hypothetical protein, mRNA | 540 | 1764 |  |
| *PM-milR-MC17* | XM_002146989.1\| nonribosomal peptide synthase, putative, mRNA | 3631 | 16992 |  |
| *PM-milR-MC17* | XM_002147001.1\| mitochondrial cation transporter, putative, mRNA | 446 | 1196 |  |
| *PM-milR-MC17* | XM_002147233.1\| multidrug resistance protein fnx1, putative, mRNA | 292 | 1765 |  |
| *PM-milR-MC17* | XM_002147333.1\| transcriptional regulator (Cti6), putative, mRNA | 905 | 2310 |  |
| *PM-milR-MC17* | XM_002147343.1\| conserved hypothetical protein, mRNA | 532 | 2055 |  |
| *PM-milR-MC17* | XM_002147354.1\| trehalose-phosphate synthase/phosphatase complex subunit Tps1, putative, mRNA | 2115 | 2240 |  |
| *PM-milR-MC17* | XM_002147356.1\| AAA family ATPase, putative, mRNA | 107 | 2598 |  |
| *PM-milR-MC17* | XM_002147366.1\| stress response transcription factor SrrA/Skn7, putative, mRNA | 196 | 1917 |  |
| *PM-milR-MC17* | XM_002147383.1\| triglyceride lipase-cholesterol esterase, putative, mRNA | 478 | 1828 |  |
| *PM-milR-MC17* | XM_002147384.1\| triglyceride lipase-cholesterol esterase, putative, mRNA | 552 | 1902 |  |
| *PM-milR-MC17* | XM_002147438.1\| alpha-1,6-mannosyltransferase subunit (Och1), putative, mRNA | 508 | 1931 |  |
| *PM-milR-MC17* | XM_002147472.1\| MAP kinase MpkA, mRNA | 1638 | 1976 |  |
| *PM-milR-MC17* | XM_002147550.1\| cytochrome c oxidase subunit Va, putative, mRNA | 119 | 813 |  |
| *PM-milR-MC17* | XM_002147744.1\| mitochondrial export translocase Oxa1, putative, mRNA | 1574 | 1906 |  |
| *PM-milR-MC17* | XM_002147775.1\| signal recognition particle protein SRP54, mRNA | 107 | 1614 |  |
| *PM-milR-MC17* | XM_002147794.1\| AP-1 adaptor complex subunit mu, putative, mRNA | 711 | 2751 |  |
| *PM-milR-MC17* | XM_002147911.1\| alcohol dehydrogenase, putative, mRNA | 659 | 1223 |  |
| *PM-milR-MC17* | XM_002148132.1\| 40S ribosomal protein S3Ae, mRNA | 452 | 1170 |  |
| *PM-milR-MC17* | XM_002148177.1\| betaine aldehyde dehydrogenase (BadH), putative, mRNA | 961 | 1497 |  |
| *PM-milR-MC17* | XM_002148297.1\| cytochrome P450 phenylacetate 2-hydroxylase, putative, mRNA | 1033 | 1883 |  |
| *PM-milR-MC17* | XM_002148343.1\| phosphoglycerate mutase family protein, putative, mRNA | 29 | 525 |  |
| *PM-milR-MC17* | XM_002148414.1\| glycogen debranching enzyme Gdb1, putative, mRNA | 3900 | 4741 |  |
| *PM-milR-MC17* | XM_002148500.1\| glucooligosaccharide oxidase, putative, mRNA | 1346 | 1578 |  |
| *PM-milR-MC17* | XM_002148546.1\| cytosolic phospholipase A2, putative, mRNA | 462 | 2223 |  |
| *PM-milR-MC17* | XM_002148648.1\| hypothetical protein, mRNA | 221 | 2074 |  |
| *PM-milR-MC17* | XM_002148673.1\| malate dehydrogenase, NAD-dependent, mRNA | 745 | 1169 |  |
| *PM-milR-MC17* | XM_002148740.1\| mitochondrial large ribosomal subunit protein L16, putative, mRNA | 856 | 956 |  |
| *PM-milR-MC17* | XM_002148752.1\| glucan endo-1,3-alpha-glucosidase agn1 precursor, putative, mRNA | 167 | 1850 |  |
| *PM-milR-MC17* | XM_002148871.1\| conserved hypothetical protein, mRNA | 815 | 1375 |  |
| *PM-milR-MC17* | XM_002148882.1\| COPII vesicles protein Yip3, putative, mRNA | 411 | 2067 |  |
| *PM-milR-MC17* | XM_002149120.1\| cytokinesis regulator (Byr4), putative, mRNA | 789 | 3552 |  |
| *PM-milR-MC17* | XM_002149188.1\| C6 transcription factor, putative, mRNA | 1680 | 2103 |  |
| *PM-milR-MC17* | XM_002149215.1\| conserved hypothetical protein, mRNA | 382 | 881 |  |
| *PM-milR-MC17* | XM_002149253.1\| conserved hypothetical protein, mRNA | 4343 | 4600 |  |
| *PM-milR-MC17* | XM_002149273.1\| conserved hypothetical protein, mRNA | 18 | 1200 |  |
| *PM-milR-MC17* | XM_002149363.1\| conserved hypothetical protein, mRNA | 86 | 1449 |  |
| *PM-milR-MC17* | XM_002149392.1\| conserved hypothetical protein, mRNA | 805 | 3626 |  |
| *PM-milR-MC17* | XM_002149589.1\| conserved hypothetical protein, mRNA | 943 | 1374 |  |
| *PM-milR-MC17* | XM_002149771.1\| C6 finger domain protein, putative, mRNA | 1095 | 1518 |  |
| *PM-milR-MC17* | XM_002149890.1\| cell cycle control protein (Cwf22), putative, mRNA | 96 | 2406 |  |
| *PM-milR-MC17* | XM_002149958.1\| conserved hypothetical protein, mRNA | 157 | 3105 |  |
| *PM-milR-MC17* | XM_002150089.1\| amino acid transporter, putative, mRNA | 1231 | 1800 |  |
| *PM-milR-MC17* | XM_002150212.1\| ubiquitin-protein ligase (Hul4), putative, mRNA | 564 | 3735 |  |
| *PM-milR-MC17* | XM_002150301.1\| UPF0136 domain protein, mRNA | 64 | 389 |  |
| *PM-milR-MC17* | XM_002150520.1\| 60S ribosomal protein L37a, mRNA | 464 | 840 |  |
| *PM-milR-MC17* | XM_002150521.1\| 60S ribosomal protein L37a, mRNA | 468 | 844 |  |
| *PM-milR-MC17* | XM_002150522.1\| 60S ribosomal protein L37a, mRNA | 488 | 864 |  |
| *PM-milR-MC17* | XM_002150523.1\| 60S ribosomal protein L37a, mRNA | 648 | 1024 |  |
| *PM-milR-MC17* | XM_002150707.1\| conserved hypothetical protein, mRNA | 1793 | 3961 |  |
| *PM-milR-MC17* | XM_002150732.1\| conserved hypothetical protein, mRNA | 828 | 1539 |  |
| *PM-milR-MC17* | XM_002150733.1\| GPI anchored protein, putative, mRNA | 626 | 949 |  |
| *PM-milR-MC17* | XM_002150734.1\| GPI anchored protein, putative, mRNA | 629 | 952 |  |
| *PM-milR-MC17* | XM_002150788.1\| CCCH zinc finger protein, mRNA | 140 | 2238 |  |
| *PM-milR-MC17* | XM_002150807.1\| carbamoyl-phosphate synthase, large subunit, mRNA | 120 | 3652 |  |
| *PM-milR-MC17* | XM_002150827.1\| C6 finger domain protein, putative, mRNA | 1458 | 2832 |  |
| *PM-milR-MC17* | XM_002151085.1\| serine/threonine-rich protein adg2 precursor, putative, mRNA | 499 | 1413 |  |
| *PM-milR-MC17* | XM_002151222.1\| proteasome activator subunit 4, putative, mRNA | 4823 | 6537 |  |
| *PM-milR-MC17* | XM_002151246.1\| glycerol-3-phosphate acyltransferase Sct1, putative, mRNA | 1588 | 2354 |  |
| *PM-milR-MC17* | XM_002151266.1\| conserved hypothetical protein, mRNA | 200 | 1152 |  |
| *PM-milR-MC17* | XM_002151337.1\| DNA replication helicase Dna2, putative, mRNA | 4355 | 5013 |  |
| *PM-milR-MC17* | XM_002151559.1\| DEAD/DEAH box RNA helicase, mRNA | 3088 | 3742 |  |
| *PM-milR-MC17* | XM_002151790.1\| Rho GTPase activator (Bem3), putative, mRNA | 103 | 4241 |  |
| *PM-milR-MC17* | XM_002151839.1\| mitochondrial translocase complex component (Tim50), putative, mRNA | 208 | 2142 |  |
| *PM-milR-MC17* | XM_002151906.1\| SH3 domain protein, mRNA | 2646 | 4172 |  |
| *PM-milR-MC17* | XM_002151907.1\| SH3 domain protein, mRNA | 2636 | 4162 |  |
| *PM-milR-MC17* | XM_002151918.1\| cytosolic regulator Pianissimo, putative, mRNA | 1842 | 4068 |  |
| *PM-milR-MC17* | XM_002152013.1\| alpha-1,3-glucan synthase Ags2, mRNA | 3072 | 7239 |  |
| *PM-milR-MC17* | XM_002152063.1\| MFS monosaccharide transporter, putative, mRNA | 1626 | 1892 |  |
| *PM-milR-MC17* | XM_002152090.1\| amino acid transporter, putative, mRNA | 1203 | 2019 |  |
| *PM-milR-MC17* | XM_002152200.1\| conserved hypothetical protein, mRNA | 11258 | 30287 |  |
| *PM-milR-MC17* | XM_002152232.1\| conserved hypothetical protein, mRNA | 192 | 1005 |  |
| *PM-milR-MC17* | XM_002152310.1\| alcohol dehydrogenase, putative, mRNA | 188 | 1359 |  |
| *PM-milR-MC17* | XM_002152359.1\| acetolactate synthase, putative, mRNA | 183 | 1812 |  |
| *PM-milR-MC17* | XM_002152383.1\| mitochondrial carrier protein (Rim2), putative, mRNA | 392 | 1282 |  |
| *PM-milR-MC17* | XM_002152420.1\| conserved hypothetical protein, mRNA | 876 | 1221 |  |
| *PM-milR-MC17* | XM_002152478.1\| conserved hypothetical protein, mRNA | 96 | 778 |  |
| *PM-milR-MC17* | XM_002152558.1\| 20S cyclosome subunit (APC1/BimE), putative, mRNA | 1612 | 6216 |  |
| *PM-milR-MC17* | XM_002152575.1\| MAP kinase kinase Ste7, mRNA | 1167 | 2109 |  |
| *PM-milR-MC17* | XM_002152704.1\| MFS transporter, putative, mRNA | 818 | 1556 |  |
| *PM-milR-MC17* | XM_002152730.1\| high affinity methionine permease, mRNA | 38 | 1865 |  |
| *PM-milR-MC17* | XM_002153168.1\| polysaccharide synthase Cps1, putative, mRNA | 967 | 2996 |  |
| *PM-milR-MC17* | XM_002153267.1\| ABC drug exporter AtrF, mRNA | 2936 | 4605 |  |
| *PM-milR-MC17* | XM_002153332.1\| threonine synthase Thr4, putative, mRNA | 900 | 1880 |  |
| *PM-milR-MC17* | XM_002153455.1\| pyruvate dehydrogenase complex component Pdx1, putative, mRNA | 36 | 1261 |  |
| *PM-milR-MC17* | XM_002153693.1\| hypothetical protein, mRNA | 226 | 1332 |  |
| *PM-milR-MC17* | XM_002143098.1\| aspergillopepsin A precursor, putative, mRNA | 152 | 1128 |  |
| *PM-milR-MC17* | XM_002143164.1\| beta-1,4-mannosyltransferase (Alg1), putative, mRNA | 458 | 1392 |  |
| *PM-milR-MC17* | XM_002143187.1\| membrane bound C2 domain protein (vp115), putative, mRNA | 636 | 4725 |  |
| *PM-milR-MC17* | XM_002143192.1\| MFS peptide transporter Ptr2, putative, mRNA | 1731 | 2067 |  |
| *PM-milR-MC17* | XM_002143202.1\| UPF0183 domain protein, mRNA | 778 | 1736 |  |
| *PM-milR-MC17* | XM_002143225.1\| farnesyl-diphosphate farnesyltransferase, putative, mRNA | 143 | 1798 |  |
| *PM-milR-MC17* | XM_002143281.1\| glutamate carboxypeptidase Tre2, putative, mRNA | 553 | 3220 |  |
| *PM-milR-MC17* | XM_002143297.1\| acetamidase, putative, mRNA | 1457 | 1863 |  |
| *PM-milR-MC17* | XM_002143309.1\| nucleus export ATPase (Elf1), putative, mRNA | 3292 | 3700 |  |
| *PM-milR-MC17* | XM_002143339.1\| F-box domain protein, mRNA | 2269 | 2640 |  |
| *PM-milR-MC17* | XM_002143358.1\| COPII vesicle coat protein Sec16, putative, mRNA | 3838 | 5406 |  |
| *PM-milR-MC17* | XM_002143377.1\| conserved hypothetical protein, mRNA | 5742 | 6236 |  |
| *PM-milR-MC17* | XM_002143431.1\| ribonuclease P complex subunit Pop1, putative, mRNA | 109 | 2768 |  |
| *PM-milR-MC17* | XM_002143530.1\| methyltransferase, putative, mRNA | 28 | 918 |  |
| *PM-milR-MC17* | XM_002143771.1\| conserved hypothetical protein, mRNA | 372 | 2321 |  |
| *PM-milR-MC17* | XM_002143822.1\| conserved hypothetical protein, mRNA | 309 | 2415 |  |
| *PM-milR-MC17* | XM_002143869.1\| RTA1 domain protein, putative, mRNA | 979 | 1387 |  |
| *PM-milR-MC17* | XM_002143936.1\| CP2 transcription factor, putative, mRNA | 1070 | 2886 |  |
| *PM-milR-MC17* | XM_002143974.1\| ferric-chelate reductase, putative, mRNA | 340 | 2029 |  |
| *PM-milR-MC17* | XM_002144007.1\| translation elongation factor EF-1 alpha subunit , putative, mRNA | 421 | 1523 |  |
| *PM-milR-MC17* | XM_002144018.1\| BAG domain protein, mRNA | 981 | 1985 |  |
| *PM-milR-MC17* | XM_002144070.1\| tRNA-specific adenosine deaminase, putative, mRNA | 1557 | 3645 |  |
| *PM-milR-MC17* | XM_002144110.1\| conserved hypothetical protein, mRNA | 106 | 1716 |  |
| *PM-milR-MC17* | XM_002144340.1\| pentatricopeptide repeat protein, mRNA | 55 | 2313 |  |
| *PM-milR-MC17* | XM_002144368.1\| conserved hypothetical protein, mRNA | 479 | 1081 |  |
| *PM-milR-MC17* | XM_002144369.1\| conserved hypothetical protein, mRNA | 548 | 1150 |  |
| *PM-milR-MC17* | XM_002144442.1\| HLH DNA binding domain protein, putative, mRNA | 130 | 1421 |  |
| *PM-milR-MC17* | XM_002144448.1\| dipeptidyl peptidase, putative, mRNA | 2952 | 3992 |  |
| *PM-milR-MC17* | XM_002144449.1\| dipeptidyl peptidase, putative, mRNA | 2952 | 3935 |  |
| *PM-milR-MC17* | XM_002144450.1\| dipeptidyl peptidase, putative, mRNA | 2952 | 3932 |  |
| *PM-milR-MC17* | XM_002144520.1\| protein disulfide isomerase Pdi1, putative, mRNA | 1495 | 2381 |  |
| *PM-milR-MC17* | XM_002144648.1\| ATP dependent RNA helicase, putative, mRNA | 1518 | 2358 |  |
| *PM-milR-MC17* | XM_002144699.1\| cytochrome b5, putative, mRNA | 306 | 437 |  |
| *PM-milR-MC17* | XM_002144702.1\| conserved hypothetical protein, mRNA | 1319 | 3720 |  |
| *PM-milR-MC17* | XM_002144790.1\| conserved hypothetical protein, mRNA | 740 | 1703 |  |
| *PM-milR-MC17* | XM_002144820.1\| AMP dependent CoA ligase, putative, mRNA | 738 | 1887 |  |
| *PM-milR-MC17* | XM_002144829.1\| polyketide synthase, putative, mRNA | 748 | 5427 |  |
| *PM-milR-MC17* | XM_002144875.1\| conserved hypothetical protein, mRNA | 610 | 1644 |  |
| *PM-milR-MC17* | XM_002144937.1\| alcohol dehydrogenase, putative, mRNA | 389 | 1434 |  |
| *PM-milR-MC17* | XM_002145111.1\| conserved hypothetical protein, mRNA | 300 | 1116 |  |
| *PM-milR-MC17* | XM_002145411.1\| Ctr copper transporter family protein, mRNA | 610 | 1120 |  |
| *PM-milR-MC17* | XM_002145412.1\| Ctr copper transporter family protein, mRNA | 840 | 1273 |  |
| *PM-milR-MC17* | XM_002145413.1\| Ctr copper transporter family protein, mRNA | 610 | 1043 |  |
| *PM-milR-MC17* | XM_002145460.1\| GTPase activating protein (Gyp2), putative, mRNA | 110 | 3603 |  |
| *PM-milR-MC17* | XM_002145569.1\| conserved hypothetical protein, mRNA | 246 | 2601 |  |
| *PM-milR-MC17* | XM_002145570.1\| conserved hypothetical protein, mRNA | 246 | 2660 |  |
| *PM-milR-MC17* | XM_002145648.1\| NRPS-like enzyme, putative, mRNA | 873 | 3946 |  |
| *PM-milR-MC17* | XM_002145786.1\| conserved hypothetical protein, mRNA | 770 | 1750 |  |
| *PM-milR-MC17* | XM_002145792.1\| glutathione-S-transferase theta, GST, putative, mRNA | 37 | 657 |  |
| *PM-milR-MC17* | XM_002145796.1\| conserved hypothetical protein, mRNA | 95 | 1716 |  |
| *PM-milR-MC17* | XM_002145799.1\| GTPase activating protein (Evi5), putative, mRNA | 1124 | 2960 |  |
| *PM-milR-MC17* | XM_002145830.1\| BSD domain protein, mRNA | 535 | 1197 |  |
| *PM-milR-MC17* | XM_002145865.1\| beta-mannosidase, mRNA | 2426 | 2862 |  |
| *PM-milR-MC17* | XM_002145891.1\| proline-specific permease, putative, mRNA | 1314 | 1650 |  |
| *PM-milR-MC17* | XM_002146007.1\| conserved hypothetical protein, mRNA | 61 | 1215 |  |
| *PM-milR-MC17* | XM_002146054.1\| BTB/POZ domain protein, mRNA | 313 | 1507 |  |
| *PM-milR-MC17* | XM_002146065.1\| hypothetical protein, mRNA | 16 | 174 |  |
| *PM-milR-MC17* | XM_002146079.1\| polyketide synthase, putative, mRNA | 896 | 7095 |  |
| *PM-milR-MC17* | XM_002146303.1\| protein kinase C substrate, putative, mRNA | 351 | 1985 |  |
| *PM-milR-MC17* | XM_002146307.1\| conserved hypothetical protein, mRNA | 521 | 1337 |  |
| *PM-milR-MC17* | XM_002146345.1\| conserved hypothetical protein, mRNA | 336 | 3751 |  |
| *PM-milR-MC17* | XM_002146346.1\| C2H2 zinc finger protein, mRNA | 303 | 3103 |  |
| *PM-milR-MC17* | XM_002146367.1\| phosphatidylinositol 4-kinase (STT4), putative, mRNA | 266 | 7873 |  |
| *PM-milR-MC17* | XM_002146399.1\| conserved hypothetical protein, mRNA | 735 | 1581 |  |
| *PM-milR-MC17* | XM_002146433.1\| C6 transcription factor, putative, mRNA | 161 | 2103 |  |
| *PM-milR-MC17* | XM_002146461.1\| involucrin repeat protein, mRNA | 17201 | 18527 |  |
| *PM-milR-MC17* | XM_002146469.1\| O-methylsterigmatocystin oxidoreductase, putative, mRNA | 1441 | 1917 |  |
| *PM-milR-MC17* | XM_002146752.1\| ribonucleotide reductase small subunit RnrA, putative, mRNA | 209 | 1621 |  |
| *PM-milR-MC17* | XM_002146935.1\| conserved hypothetical protein, mRNA | 6 | 1658 |  |
| *PM-milR-MC17* | XM_002146956.1\| conserved hypothetical protein, mRNA | 318 | 1062 |  |
| *PM-milR-MC17* | XM_002147089.1\| C6 transcription factor, putative, mRNA | 189 | 2248 |  |
| *PM-milR-MC17* | XM_002147090.1\| C6 transcription factor, putative, mRNA | 150 | 2209 |  |
| *PM-milR-MC17* | XM_002147183.1\| aminotransferase family protein (LolT), putative, mRNA | 1218 | 1750 |  |
| *PM-milR-MC17* | XM_002147259.1\| cytosolic large ribosomal subunit protein L7A, mRNA | 712 | 1157 |  |
| *PM-milR-MC17* | XM_002147335.1\| Woronin body protein HexA, putative, mRNA | 1505 | 1919 |  |
| *PM-milR-MC17* | XM_002147461.1\| cell wall glucanase (Scw4), putative, mRNA | 1616 | 2188 |  |
| *PM-milR-MC17* | XM_002147533.1\| conserved hypothetical protein, mRNA | 1202 | 2027 |  |
| *PM-milR-MC17* | XM_002147541.1\| AMID-like mitochondrial oxidoreductase, putative, mRNA | 874 | 1311 |  |
| *PM-milR-MC17* | XM_002147560.1\| histone acetylase complex subunit Paf400, putative, mRNA | 1460 | 11574 |  |
| *PM-milR-MC17* | XM_002147624.1\| DUF907 domain protein, mRNA | 69 | 2827 |  |
| *PM-milR-MC17* | XM_002147634.1\| developmental regulatory protein WetA, mRNA | 1173 | 4366 |  |
| *PM-milR-MC17* | XM_002147660.1\| LCCL domain protein, mRNA | 1060 | 2250 |  |
| *PM-milR-MC17* | XM_002147749.1\| transcription initiation factor TFIID complex 60 kDa subunit, mRNA | 156 | 1793 |  |
| *PM-milR-MC17* | XM_002147836.1\| dolichyl-phosphate beta-glucosyltransferase, putative, mRNA | 223 | 1576 |  |
| *PM-milR-MC17* | XM_002147883.1\| conserved hypothetical protein, mRNA | 1012 | 1494 |  |
| *PM-milR-MC17* | XM_002147887.1\| WD repeat protein, mRNA | 375 | 2486 |  |
| *PM-milR-MC17* | XM_002147901.1\| importin beta-5 subunit, putative, mRNA | 151 | 3232 |  |
| *PM-milR-MC17* | XM_002147944.1\| conserved hypothetical protein, mRNA | 930 | 1619 |  |
| *PM-milR-MC17* | XM_002147961.1\| SNF2 family helicase/ATPase (Ino80), putative, mRNA | 2844 | 8085 |  |
| *PM-milR-MC17* | XM_002148028.1\| aminopeptidase, putative, mRNA | 992 | 2316 |  |
| *PM-milR-MC17* | XM_002148057.1\| conserved hypothetical protein, mRNA | 1222 | 1941 |  |
| *PM-milR-MC17* | XM_002148071.1\| cytochrome P450, putative, mRNA | 398 | 1641 |  |
| *PM-milR-MC17* | XM_002148085.1\| Golgi membrane protein, putative, mRNA | 694 | 987 |  |
| *PM-milR-MC17* | XM_002148160.1\| calcium channel subunit Mid1, mRNA | 183 | 2072 |  |
| *PM-milR-MC17* | XM_002148173.1\| hypothetical protein, mRNA | 151 | 753 |  |
| *PM-milR-MC17* | XM_002148186.1\| conserved hypothetical protein, mRNA | 67 | 2550 |  |
| *PM-milR-MC17* | XM_002148196.1\| mitochondrial Hsp70 chaperone (Ssc70), putative, mRNA | 398 | 2670 |  |
| *PM-milR-MC17* | XM_002148296.1\| aquaporin transporter, putative, mRNA | 200 | 1077 |  |
| *PM-milR-MC17* | XM_002148459.1\| aminoacyl-tRNA hydrolase, putative, mRNA | 489 | 918 |  |
| *PM-milR-MC17* | XM_002148460.1\| aminoacyl-tRNA hydrolase, putative, mRNA | 469 | 898 |  |
| *PM-milR-MC17* | XM_002148461.1\| aminoacyl-tRNA hydrolase, putative, mRNA | 469 | 1005 |  |
| *PM-milR-MC17* | XM_002148462.1\| aminoacyl-tRNA hydrolase, putative, mRNA | 575 | 1004 |  |
| *PM-milR-MC17* | XM_002148475.1\| GPI transamidase component PIG-U, putative, mRNA | 1041 | 1431 |  |
| *PM-milR-MC17* | XM_002148618.1\| transcription factor Tos4, putative, mRNA | 753 | 1952 |  |
| *PM-milR-MC17* | XM_002148698.1\| conserved hypothetical protein, mRNA | 1239 | 1913 |  |
| *PM-milR-MC17* | XM_002148892.1\| CFEM domain protein, putative, mRNA | 674 | 896 |  |
| *PM-milR-MC17* | XM_002148896.1\| endoplasmic reticulum calcium ATPase, putative, mRNA | 190 | 3456 |  |
| *PM-milR-MC17* | XM_002148922.1\| snRNP assembly factor, putative, mRNA | 1254 | 1839 |  |
| *PM-milR-MC17* | XM_002149002.1\| small nucleolar ribonucleoprotein complex subunit, putative, mRNA | 2156 | 3126 |  |
| *PM-milR-MC17* | XM_002149072.1\| aminopeptidase, putative, mRNA | 3894 | 4106 |  |
| *PM-milR-MC17* | XM_002149082.1\| conserved hypothetical protein, mRNA | 154 | 2643 |  |
| *PM-milR-MC17* | XM_002149083.1\| polyketide synthase, putative, mRNA | 344 | 6429 |  |
| *PM-milR-MC17* | XM_002149085.1\| Pumilio-family RNA binding repeat domain protein, mRNA | 46 | 2957 |  |
| *PM-milR-MC17* | XM_002149122.1\| NADH-ubiquinone oxidoreductase, subunit G, putative, mRNA | 530 | 2772 |  |
| *PM-milR-MC17* | XM_002149207.1\| glucan 1,4-alpha-glucosidase, putative, mRNA | 1534 | 1926 |  |
| *PM-milR-MC17* | XM_002149212.1\| phospholipid-transporting ATPase (DRS2), putative, mRNA | 4501 | 5154 |  |
| *PM-milR-MC17* | XM_002149254.1\| glutamate synthase Glt1, putative, mRNA | 5115 | 6784 |  |
| *PM-milR-MC17* | XM_002149282.1\| 6-phosphofructo-2-kinase 1, mRNA | 792 | 2440 |  |
| *PM-milR-MC17* | XM_002149310.1\| NADH-ubiquinone oxidoreductase 14 kDa subunit, putative, mRNA | 271 | 716 |  |
| *PM-milR-MC17* | XM_002149314.1\| 37S ribosomal protein Mrp17, mRNA | 304 | 812 |  |
| *PM-milR-MC17* | XM_002149327.1\| high-affinity nicotinic acid transporter, putative, mRNA | 358 | 1494 |  |
| *PM-milR-MC17* | XM_002149497.1\| conserved hypothetical protein, mRNA | 144 | 1693 |  |
| *PM-milR-MC17* | XM_002149526.1\| cell wall glucanase (Scw11), putative, mRNA | 604 | 2511 |  |
| *PM-milR-MC17* | XM_002149579.1\| polyketide synthase, putative, mRNA | 2727 | 5265 |  |
| *PM-milR-MC17* | XM_002149616.1\| conserved hypothetical protein, mRNA | 1232 | 1617 |  |
| *PM-milR-MC17* | XM_002149624.1\| kinesin family protein, mRNA | 2585 | 5327 |  |
| *PM-milR-MC17* | XM_002149673.1\| MFS transporter, putative, mRNA | 281 | 1443 |  |
| *PM-milR-MC17* | XM_002149674.1\| MFS transporter, putative, mRNA | 281 | 1506 |  |
| *PM-milR-MC17* | XM_002149874.1\| conserved hypothetical protein, mRNA | 149 | 1797 |  |
| *PM-milR-MC17* | XM_002149953.1\| aspartyl-tRNA synthetase, cytoplasmic, mRNA | 690 | 2856 |  |
| *PM-milR-MC17* | XM_002149960.1\| NTF2 and RRM domain protein, mRNA | 1006 | 1650 |  |
| *PM-milR-MC17* | XM_002149999.1\| probable O-sialoglycoprotein endopeptidase, mRNA | 49 | 1522 |  |
| *PM-milR-MC17* | XM_002150040.1\| DENN (AEX-3) domain protein, mRNA | 2148 | 3385 |  |
| *PM-milR-MC17* | XM_002150084.1\| 1,3-beta-glucanosyltransferase Gel2, mRNA | 790 | 1637 |  |
| *PM-milR-MC17* | XM_002150108.1\| acetyl xylan esterase (Axe1), putative, mRNA | 508 | 1117 |  |
| *PM-milR-MC17* | XM_002150141.1\| 60S ribosomal protein P0, mRNA | 792 | 1160 |  |
| *PM-milR-MC17* | XM_002150150.1\| SAM domain protein, mRNA | 198 | 2538 |  |
| *PM-milR-MC17* | XM_002150176.1\| general stress response phosphoprotein phosphatase Psr1/2, putative, mRNA | 1891 | 2627 |  |
| *PM-milR-MC17* | XM_002150199.1\| NADH-cytochrome b5 reductase, putative, mRNA | 449 | 1313 |  |
| *PM-milR-MC17* | XM_002150200.1\| NADH-cytochrome b5 reductase, putative, mRNA | 513 | 1377 |  |
| *PM-milR-MC17* | XM_002150223.1\| cyclin (Pcl1), putative, mRNA | 690 | 1758 |  |
| *PM-milR-MC17* | XM_002150296.1\| DUF726 domain protein, mRNA | 169 | 3359 |  |
| *PM-milR-MC17* | XM_002150421.1\| C2H2 transcription factor (Seb1), putative, mRNA | 340 | 2630 |  |
| *PM-milR-MC17* | XM_002150430.1\| 60S ribosomal protein L25, putative, mRNA | 464 | 1023 |  |
| *PM-milR-MC17* | XM_002150476.1\| beta-lactamase family protein, mRNA | 28 | 1302 |  |
| *PM-milR-MC17* | XM_002150517.1\| TBC domain protein, putative, mRNA | 1731 | 2609 |  |
| *PM-milR-MC17* | XM_002150525.1\| Na/K ATPase alpha 1 subunit, putative, mRNA | 830 | 3502 |  |
| *PM-milR-MC17* | XM_002150625.1\| amino acid permease, putative, mRNA | 1233 | 1915 |  |
| *PM-milR-MC17* | XM_002150643.1\| GPI-anchored cell wall beta-1,3-endoglucanase EglC, mRNA | 761 | 1738 |  |
| *PM-milR-MC17* | XM_002150702.1\| conserved hypothetical protein, mRNA | 1322 | 3694 |  |
| *PM-milR-MC17* | XM_002150714.1\| conserved hypothetical protein, mRNA | 864 | 1604 |  |
| *PM-milR-MC17* | XM_002150793.1\| C2H2 finger domain protein, putative, mRNA | 885 | 3779 |  |
| *PM-milR-MC17* | XM_002150814.1\| origin recognition complex subunit 3, putative, mRNA | 1577 | 2127 |  |
| *PM-milR-MC17* | XM_002150889.1\| conserved hypothetical protein, mRNA | 627 | 837 |  |
| *PM-milR-MC17* | XM_002150900.1\| conserved hypothetical protein, mRNA | 364 | 1785 |  |
| *PM-milR-MC17* | XM_002150971.1\| hypothetical protein, mRNA | 20 | 1335 |  |
| *PM-milR-MC17* | XM_002151137.1\| polyubiquitin UbiD/Ubi4, putative, mRNA | 308 | 1186 |  |
| *PM-milR-MC17* | XM_002151138.1\| C6 finger domain protein, putative, mRNA | 2158 | 3173 |  |
| *PM-milR-MC17* | XM_002151210.1\| NADH-ubiquinone oxidoreductase, subunit F, putative, mRNA | 65 | 1645 |  |
| *PM-milR-MC17* | XM_002151261.1\| MFS monocarboxylate transporter, putative, mRNA | 1247 | 1639 |  |
| *PM-milR-MC17* | XM_002151273.1\| conserved hypothetical protein, mRNA | 2728 | 2833 |  |
| *PM-milR-MC17* | XM_002151282.1\| conserved hypothetical protein, mRNA | 1041 | 3212 |  |
| *PM-milR-MC17* | XM_002151306.1\| tubulin-specific chaperone, putative, mRNA | 1553 | 1880 |  |
| *PM-milR-MC17* | XM_002151316.1\| anthranilate phosphoribosyltransferase, putative, mRNA | 492 | 1406 |  |
| *PM-milR-MC17* | XM_002151322.1\| endosomal peripheral membrane protein (Mon2), putative, mRNA | 2047 | 5214 |  |
| *PM-milR-MC17* | XM_002151366.1\| MFS multidrug transporter, putative, mRNA | 1924 | 2704 |  |
| *PM-milR-MC17* | XM_002151385.1\| ammonium transporter (Mep2), putative, mRNA | 1227 | 1870 |  |
| *PM-milR-MC17* | XM_002151386.1\| MAP kinase kinase kinase SskB, putative, mRNA | 1892 | 4344 |  |
| *PM-milR-MC17* | XM_002151550.1\| C2H2 transcription factor (Egr2), putative, mRNA | 205 | 624 |  |
| *PM-milR-MC17* | XM_002151572.1\| hypothetical protein, mRNA | 273 | 951 |  |
| *PM-milR-MC17* | XM_002151672.1\| eukaryotic translation initiation factor 3 subunit EifCk, putative, mRNA | 2 | 1076 |  |
| *PM-milR-MC17* | XM_002151718.1\| conserved hypothetical protein, mRNA | 100 | 714 |  |
| *PM-milR-MC17* | XM_002151733.1\| conserved hypothetical protein, mRNA | 123 | 318 |  |
| *PM-milR-MC17* | XM_002151737.1\| conserved hypothetical protein, mRNA | 643 | 1268 |  |
| *PM-milR-MC17* | XM_002151779.1\| SET domain protein, mRNA | 2016 | 4099 |  |
| *PM-milR-MC17* | XM_002151780.1\| conserved hypothetical protein, mRNA | 599 | 1904 |  |
| *PM-milR-MC17* | XM_002151811.1\| phosphomannomutase (Sec53), putative, mRNA | 342 | 1163 |  |
| *PM-milR-MC17* | XM_002151825.1\| sulfate transporter, putative, mRNA | 113 | 1361 |  |
| *PM-milR-MC17* | XM_002151904.1\| 40S ribosomal protein S17, putative, mRNA | 1541 | 1660 |  |
| *PM-milR-MC17* | XM_002151945.1\| C2H2 transcription factor (Con7), putative, mRNA | 173 | 2435 |  |
| *PM-milR-MC17* | XM_002151946.1\| C2H2 transcription factor (Con7), putative, mRNA | 173 | 2291 |  |
| *PM-milR-MC17* | XM_002151947.1\| C2H2 transcription factor (Con7), putative, mRNA | 173 | 2300 |  |
| *PM-milR-MC17* | XM_002151948.1\| C2H2 transcription factor (Con7), putative, mRNA | 173 | 2348 |  |
| *PM-milR-MC17* | XM_002151949.1\| C2H2 transcription factor (Con7), putative, mRNA | 173 | 2421 |  |
| *PM-milR-MC17* | XM_002152107.1\| Mob1 family protein, mRNA | 211 | 1946 |  |
| *PM-milR-MC17* | XM_002152116.1\| dimethyladenosine transferase, mRNA | 512 | 1329 |  |
| *PM-milR-MC17* | XM_002152303.1\| glycosyl transferase, putative, mRNA | 2319 | 3253 |  |
| *PM-milR-MC17* | XM_002152305.1\| hypothetical protein, mRNA | 584 | 2271 |  |
| *PM-milR-MC17* | XM_002152350.1\| conserved hypothetical protein, mRNA | 1541 | 1752 |  |
| *PM-milR-MC17* | XM_002152567.1\| conserved hypothetical protein, mRNA | 1042 | 2946 |  |
| *PM-milR-MC17* | XM_002152643.1\| cell cycle protein kinase, putative, mRNA | 370 | 1984 |  |
| *PM-milR-MC17* | XM_002152713.1\| alpha,alpha-trehalose glucohydrolase TreA/Ath1, mRNA | 1086 | 3120 |  |
| *PM-milR-MC17* | XM_002152720.1\| MFS monocarboxylate transporter, putative, mRNA | 25 | 1317 |  |
| *PM-milR-MC17* | XM_002152732.1\| beta-1,6-glucanase Neg1, putative, mRNA | 970 | 1500 |  |
| *PM-milR-MC17* | XM_002152734.1\| DUF1212 domain membrane protein, mRNA | 2250 | 2484 |  |
| *PM-milR-MC17* | XM_002152745.1\| H /K ATPase alpha subunit, putative, mRNA | 318 | 3482 |  |
| *PM-milR-MC17* | XM_002152776.1\| plasma membrane channel protein (Aqy1), putative, mRNA | 2140 | 2412 |  |
| *PM-milR-MC17* | XM_002152965.1\| hypothetical protein, mRNA | 464 | 3516 |  |
| *PM-milR-MC17* | XM_002152981.1\| conserved hypothetical protein, mRNA | 769 | 1191 |  |
| *PM-milR-MC17* | XM_002153056.1\| ABC transporter, putative, mRNA | 24 | 4865 |  |
| *PM-milR-MC17* | XM_002153098.1\| molybdopterin synthase small subunit CnxG, mRNA | 17 | 279 |  |
| *PM-milR-MC17* | XM_002153100.1\| conserved hypothetical protein, mRNA | 266 | 3573 |  |
| *PM-milR-MC17* | XM_002153293.1\| hydrolase, alpha/beta fold family protein, mRNA | 1601 | 1677 |  |
| *PM-milR-MC17* | XM_002153322.1\| MFS transporter, putative, mRNA | 936 | 1987 |  |
| *PM-milR-MC17* | XM_002153369.1\| short-chain dehydrogenases/reductase, putative, mRNA | 447 | 1032 |  |
| *PM-milR-MC17* | XM_002153408.1\| sucrose transport protein, putative, mRNA | 921 | 1899 |  |
| *PM-milR-MC17* | XM_002153438.1\| peroxisome biosynthesis protein (PAS8/Peroxin-6), putative, mRNA | 1662 | 4596 |  |
| *PM-milR-MC17* | XM_002153521.1\| FHA domain protein, mRNA | 434 | 2379 |  |
| *PM-milR-MC17* | XM_002153548.1\| RNA polymerase II general transcription and DNA repair factor TFIIH component Tfb5, putative, mRNA | 46 | 1151 |  |
| *PM-milR-MC17* | XM_002153578.1\| conserved hypothetical protein, mRNA | 835 | 2577 |  |
| *PM-milR-MC17* | XM_002153589.1\| MFS transporter of unkown specificity, mRNA | 1049 | 1633 |  |
| *PM-milR-MC17* | XM_002153653.1\| hypothetical protein, mRNA | 28 | 399 |  |
| *PM-milR-MC17* | XM_002153657.1\| conserved hypothetical protein, mRNA | 682 | 1575 |  |
| *PM-milR-MC17* | XM_002153659.1\| hypothetical protein, mRNA | 874 | 2855 |  |
| *PM-milR-MC17* | XM_002153678.1\| reverse transcriptase, putative, mRNA | 520 | 1888 |  |
| *PM-milR-YC1* | XM_002143780.1\| conserved hypothetical protein, mRNA | 312 | 999 |  |
| *PM-milR-YC1* | XM_002153194.1\| RNA methyltransferase, putative, mRNA | 514 | 1300 |  |
| *PM-milR-YC1* | XM_002153466.1\| alpha-mannosidase, mRNA | 425 | 3276 |  |
| *PM-milR-YC2* | XM_002145664.1\| conserved hypothetical protein, mRNA | 272 | 2499 |  |
| *PM-milR-YC3* | XM_002145038.1\| DUF1264 domain protein, mRNA | 982 | 1155 |  |
| *PM-milR-YC3* | XM_002152662.1\| histone H2B, mRNA | 616 | 1305 |  |
| *PM-milR-YC3* | XM_002144322.1\| ubiquitin fusion degradation protein UfdB, putative, mRNA | 482 | 3192 |  |
| *PM-milR-YC3* | XM_002145038.1\| DUF1264 domain protein, mRNA | 981 | 1155 | perfectly complementary |
| *PM-milR-YC4* | XM_002143303.1\| hypothetical protein, mRNA | 856 | 956 |  |
| *PM-milR-YC4* | XM_002149698.1\| protein kinase, putative, mRNA | 730 | 3773 |  |
| *PM-milR-YC5* | XM_002143637.1\| stress response protein Nst1, putative, mRNA | 1791 | 4314 |  |
| *PM-milR-YC5* | XM_002151779.1\| SET domain protein, mRNA | 2835 | 4099 |  |
| *PM-milR-YC5* | XM_002143393.1\| chromosome segregation protein Cse1, putative, mRNA | 2761 | 3411 |  |
| *PM-milR-YC5* | XM_002143583.1\| peroxisomal dehydratase, putative, mRNA | 1377 | 1779 |  |
| *PM-milR-YC5* | XM_002144121.1\| DUF1295 domain protein, mRNA | 801 | 1157 |  |
| *PM-milR-YC5* | XM_002146418.1\| nonribosomal siderophore peptide synthase, putative, mRNA | 2029 | 15318 |  |
| *PM-milR-YC5* | XM_002146580.1\| ubiquitin hydrolase L3, mRNA | 690 | 852 |  |
| *PM-milR-YC5* | XM_002147049.1\| conserved hypothetical protein, mRNA | 1457 | 4671 |  |
| *PM-milR-YC5* | XM_002148515.1\| conserved hypothetical protein, mRNA | 1388 | 4494 |  |
| *PM-milR-YC5* | XM_002148861.1\| N,N-dimethylglycine oxidase, mRNA | 1248 | 1479 |  |
| *PM-milR-YC5* | XM_002148885.1\| beta-galactosidase, putative, mRNA | 2968 | 3000 |  |
| *PM-milR-YC5* | XM_002149766.1\| conserved hypothetical protein, mRNA | 1457 | 4431 |  |
| *PM-milR-YC5* | XM_002150658.1\| conserved hypothetical protein, mRNA | 413 | 2346 |  |
| *PM-milR-YC5* | XM_002151109.1\| transcription factor, putative, mRNA | 1895 | 2605 |  |
| *PM-milR-YC5* | XM_002152788.1\| conserved hypothetical protein, mRNA | 1668 | 5236 |  |
| *PM-milR-YC6* | XM_002144523.1\| heterogeneous nuclear ribonucleoprotein HRP1, mRNA | 1324 | 1365 |  |
| *PM-milR-YC6* | XM_002145827.1\| conserved hypothetical protein, mRNA | 693 | 2073 |  |
| *PM-milR-YC6* | XM_002146225.1\| nitrite reductase NiiA, mRNA | 680 | 3294 |  |
| *PM-milR-YC6* | XM_002146859.1\| sugar 1,4-lactone oxidase, putative, mRNA | 1574 | 1890 |  |
| *PM-milR-YC6* | XM_002146962.1\| nonribosomal peptide synthase, putative, mRNA | 7019 | 16626 |  |
| *PM-milR-YC6* | XM_002147380.1\| cytochrome c oxidase assembly protein (Pet191), putative, mRNA | 818 | 1220 |  |
| *PM-milR-YC6* | XM_002147381.1\| cytochrome c oxidase assembly protein (Pet191), putative, mRNA | 976 | 1378 |  |
| *PM-milR-YC6* | XM_002148879.1\| endoplasmic reticulum DnaJ domain protein Erj5, putative, mRNA | 1125 | 1330 |  |
| *PM-milR-YC6* | XM_002151161.1\| cell cycle regulatory protein, putative, mRNA | 1602 | 2787 |  |
| *PM-milR-YC6* | XM_002151222.1\| proteasome activator subunit 4, putative, mRNA | 3341 | 6537 |  |
| *PM-milR-YC6* | XM_002152059.1\| hypothetical protein, mRNA | 1289 | 1344 |  |
| *PM-milR-YC6* | XM_002153707.1\| RNA interference and gene silencing protein (Qde2), putative, mRNA | 455 | 3892 |  |
| *PM-milR-YC6* | XM_002143093.1\| hypothetical protein, mRNA | 1612 | 2094 |  |
| *PM-milR-YC6* | XM_002143101.1\| conserved hypothetical protein, mRNA | 1309 | 1551 |  |
| *PM-milR-YC6* | XM_002143799.1\| myo-inositol-phosphate synthase, putative, mRNA | 1443 | 2312 |  |
| *PM-milR-YC6* | XM_002143906.1\| t-complex protein 1, gamma subunit (Cct3), putative, mRNA | 1341 | 1898 |  |
| *PM-milR-YC6* | XM_002144564.1\| DNA replication licensing factor Mcm4, putative, mRNA | 755 | 3083 |  |
| *PM-milR-YC6* | XM_002144644.1\| NRPS-like enzyme, putative, mRNA | 2741 | 3126 |  |
| *PM-milR-YC6* | XM_002144825.1\| transesterase (LovD), putative, mRNA | 945 | 1337 |  |
| *PM-milR-YC6* | XM_002145209.1\| KH domain protein, mRNA | 369 | 3491 |  |
| *PM-milR-YC6* | XM_002145210.1\| chitin synthase ChsE, mRNA | 4640 | 5950 |  |
| *PM-milR-YC6* | XM_002145297.1\| conserved hypothetical protein, mRNA | 225 | 2658 |  |
| *PM-milR-YC6* | XM_002145756.1\| polyketide synthase, putative, mRNA | 1146 | 6516 |  |
| *PM-milR-YC6* | XM_002146024.1\| glutathione oxidoreductase Glr1, putative, mRNA | 1248 | 1942 |  |
| *PM-milR-YC6* | XM_002146120.1\| oligopeptide transporter, OPT family, putative, mRNA | 324 | 2562 |  |
| *PM-milR-YC6* | XM_002146124.1\| conserved hypothetical protein, mRNA | 2221 | 3000 |  |
| *PM-milR-YC6* | XM_002146186.1\| geranylgeranyl diphosphate synthase, mRNA | 419 | 2137 |  |
| *PM-milR-YC6* | XM_002146263.1\| TMEM1 family protein, putative, mRNA | 1032 | 4496 |  |
| *PM-milR-YC6* | XM_002146356.1\| condensin complex component cnd2, mRNA | 333 | 2772 |  |
| *PM-milR-YC6* | XM_002146368.1\| cell-cycle checkpoint protein kinase, putative, mRNA | 1115 | 2416 |  |
| *PM-milR-YC6* | XM_002146617.1\| sister chromatid separation protein (Src1), putative, mRNA | 1993 | 2301 |  |
| *PM-milR-YC6* | XM_002147122.1\| hypothetical protein, mRNA | 1540 | 2676 |  |
| *PM-milR-YC6* | XM_002147125.1\| conserved hypothetical protein, mRNA | 286 | 1065 |  |
| *PM-milR-YC6* | XM_002147612.1\| conserved hypothetical protein, mRNA | 1031 | 1503 |  |
| *PM-milR-YC6* | XM_002147752.1\| hypothetical protein, mRNA | 1272 | 1404 |  |
| *PM-milR-YC6* | XM_002147840.1\| aldo-keto reductase, putative, mRNA | 565 | 1158 |  |
| *PM-milR-YC6* | XM_002148534.1\| reverse transcriptase, putative, mRNA | 476 | 3789 |  |
| *PM-milR-YC6* | XM_002148901.1\| enolase/allergen Asp F 22, mRNA | 62 | 1482 |  |
| *PM-milR-YC6* | XM_002149014.1\| lipase 8 precursor, putative, mRNA | 1173 | 1329 |  |
| *PM-milR-YC6* | XM_002149058.1\| RNA binding protein Nrd1, putative, mRNA | 1188 | 2363 |  |
| *PM-milR-YC6* | XM_002149059.1\| RNA binding protein Nrd1, putative, mRNA | 1188 | 1861 |  |
| *PM-milR-YC6* | XM_002149171.1\| pyruvate dehydrogenase E1 component alpha subunit, putative, mRNA | 1026 | 1410 |  |
| *PM-milR-YC6* | XM_002149622.1\| MFS lactose permease, putative, mRNA | 1605 | 1641 |  |
| *PM-milR-YC6* | XM_002149753.1\| hypothetical protein, mRNA | 702 | 2052 |  |
| *PM-milR-YC6* | XM_002149763.1\| conserved hypothetical protein, mRNA | 236 | 1203 |  |
| *PM-milR-YC6* | XM_002149779.1\| hypothetical protein, mRNA | 667 | 735 |  |
| *PM-milR-YC6* | XM_002149914.1\| conserved hypothetical protein, mRNA | 556 | 741 |  |
| *PM-milR-YC6* | XM_002149965.1\| short-chain dehydrogenase, putative, mRNA | 181 | 999 |  |
| *PM-milR-YC6* | XM_002150429.1\| mRNA splicing protein (Prp39), putative, mRNA | 955 | 1989 |  |
| *PM-milR-YC6* | XM_002150542.1\| GPR/FUN34 family protein, mRNA | 708 | 1460 |  |
| *PM-milR-YC6* | XM_002150699.1\| pre-mRNA splicing helicase, putative, mRNA | 1228 | 6753 |  |
| *PM-milR-YC6* | XM_002150730.1\| alpha-N-acetylglucosaminidase, putative, mRNA | 523 | 2797 |  |
| *PM-milR-YC6* | XM_002150808.1\| conserved hypothetical protein, mRNA | 1387 | 2004 |  |
| *PM-milR-YC6* | XM_002151562.1\| conserved hypothetical protein, mRNA | 596 | 747 |  |
| *PM-milR-YC6* | XM_002152173.1\| hypothetical protein, mRNA | 181 | 939 |  |
| *PM-milR-YC6* | XM_002152267.1\| zinc-binding oxidoreductase ToxD, putative, mRNA | 285 | 1399 |  |
| *PM-milR-YC6* | XM_002152358.1\| conserved hypothetical protein, mRNA | 830 | 1545 |  |
| *PM-milR-YC6* | XM_002152698.1\| bZIP transcription factor HacA, mRNA | 392 | 1629 |  |
| *PM-milR-YC6* | XM_002152778.1\| conserved hypothetical protein, mRNA | 743 | 1633 |  |
| *PM-milR-YC6* | XM_002152806.1\| MFS sugar transporter, putative, mRNA | 1628 | 2274 |  |
| *PM-milR-YC6* | XM_002152917.1\| hypothetical protein, mRNA | 1390 | 2859 |  |
| *PM-milR-YC6* | XM_002153137.1\| mRNA binding post-transcriptional regulator (Csx1), putative, mRNA | 414 | 1875 |  |
| *PM-milR-YC6* | XM_002153254.1\| conserved hypothetical protein, mRNA | 1575 | 1644 |  |
| *PM-milR-YC6* | XM_002153336.1\| Coatomer subunit alpha, putative, mRNA | 2002 | 4070 |  |
| *PM-milR-YC6* | XM_002153601.1\| protein disulfide isomerase, putative, mRNA | 369 | 1294 |  |
| *PM-milR-YC6* | XM_002153619.1\| DNA helicase recq, putative, mRNA | 982 | 4695 |  |
| *PM-milR-YC7* | XM_002143822.1\| conserved hypothetical protein, mRNA | 5 | 2415 |  |
| *PM-milR-YC7* | XM_002143824.1\| dihydroxy acid dehydratase Ilv3, putative, mRNA | 21 | 1925 |  |
| *PM-milR-YC7* | XM_002145052.1\| Apc13 domain protein, mRNA | 352 | 588 |  |
| *PM-milR-YC7* | XM_002146490.1\| conserved hypothetical protein, mRNA | 2234 | 2374 |  |
| *PM-milR-YC7* | XM_002146809.1\| conserved hypothetical protein, mRNA | 236 | 3447 |  |
| *PM-milR-YC7* | XM_002146810.1\| conserved hypothetical protein, mRNA | 236 | 3546 |  |
| *PM-milR-YC7* | XM_002146914.1\| conserved hypothetical protein, mRNA | 871 | 1207 |  |
| *PM-milR-YC7* | XM_002147290.1\| protein transport protein Sec24, putative, mRNA | 65 | 2816 |  |
| *PM-milR-YC7* | XM_002148448.1\| phosphoserine aminotransferase, mRNA | 1268 | 1585 |  |
| *PM-milR-YC7* | XM_002149613.1\| cytochrome P450, putative, mRNA | 239 | 501 |  |
| *PM-milR-YC7* | XM_002150352.1\| WD repeat protein, mRNA | 3421 | 4044 |  |
| *PM-milR-YC7* | XM_002150581.1\| EF hand domain protein, mRNA | 1781 | 3043 |  |
| *PM-milR-YC7* | XM_002151274.1\| GINS complex subunit Psf3, putative, mRNA | 793 | 880 |  |
| *PM-milR-YC7* | XM_002151275.1\| GINS complex subunit Psf3, putative, mRNA | 855 | 942 |  |
| *PM-milR-YC7* | XM_002152109.1\| conserved hypothetical protein, mRNA | 357 | 1928 |  |
| *PM-milR-YC7* | XM_002143209.1\| conserved hypothetical protein, mRNA | 243 | 954 |  |
| *PM-milR-YC7* | XM_002144014.1\| conserved hypothetical protein, mRNA | 872 | 1389 |  |
| *PM-milR-YC7* | XM_002144498.1\| ubiquitin hydrolase, putative, mRNA | 2432 | 2555 |  |
| *PM-milR-YC7* | XM_002144574.1\| translation initiation protein Sua5, mRNA | 1024 | 1506 |  |
| *PM-milR-YC7* | XM_002144776.1\| DNA repair and transcription protein (Xab2), putative, mRNA | 2436 | 2769 |  |
| *PM-milR-YC7* | XM_002145143.1\| conserved hypothetical protein, mRNA | 2255 | 9215 |  |
| *PM-milR-YC7* | XM_002145188.1\| aromatic aminotransferase Aro8, putative, mRNA | 1573 | 1908 |  |
| *PM-milR-YC7* | XM_002145227.1\| conserved hypothetical protein, mRNA | 1016 | 1092 |  |
| *PM-milR-YC7* | XM_002146750.1\| WD repeat protein, mRNA | 185 | 2293 |  |
| *PM-milR-YC7* | XM_002147163.1\| conserved hypothetical protein, mRNA | 456 | 1598 |  |
| *PM-milR-YC7* | XM_002147859.1\| pyridoxine biosynthesis protein, mRNA | 173 | 1179 |  |
| *PM-milR-YC7* | XM_002148040.1\| alanine racemase family protein, putative, mRNA | 815 | 1015 |  |
| *PM-milR-YC7* | XM_002149657.1\| alpha-1,6-mannosyltransferase subunit, putative, mRNA | 5388 | 5838 |  |
| *PM-milR-YC7* | XM_002150001.1\| conserved hypothetical protein, mRNA | 895 | 1507 |  |
| *PM-milR-YC7* | XM_002150338.1\| conserved hypothetical protein, mRNA | 139 | 1113 |  |
| *PM-milR-YC7* | XM_002150449.1\| monocarboxylate permease, putative, mRNA | 309 | 1200 |  |
| *PM-milR-YC7* | XM_002150790.1\| ABC multidrug transporter, putative, mRNA | 2402 | 5003 |  |
| *PM-milR-YC7* | XM_002151401.1\| calcium channel subunit Cch1, mRNA | 543 | 6791 |  |
| *PM-milR-YC7* | XM_002152324.1\| conserved hypothetical protein, mRNA | 1989 | 2386 |  |
| *PM-milR-YC7* | XM_002152713.1\| alpha,alpha-trehalose glucohydrolase TreA/Ath1, mRNA | 489 | 3120 |  |
| *PM-milR-YC7* | XM_002152722.1\| chlorohydrolase family protein, mRNA | 145 | 1564 |  |
| *PM-milR-YC7* | XM_002153429.1\| hypothetical protein, mRNA | 530 | 714 |  |
